# The Lack of the TetR-Like Repressor Gene *BCG*_*2177c* (*Rv2160A*) May Help Mycobacteria Overcome Intracellular Redox Stress and Survive Longer Inside Macrophages When Surrounded by a Lipid Environment

**DOI:** 10.3389/fcimb.2022.907890

**Published:** 2022-07-07

**Authors:** Lázaro García-Morales, Patricia Del Portillo, Juan M. Anzola, Miguel A. Ares, Addy C. Helguera-Repetto, Jorge F. Cerna-Cortes, Alfonso Méndez-Tenorio, María J. García, Isabel Otal, Carlos Martín, Jorge A. Gonzalez-y-Merchand, Sandra Rivera-Gutiérrez

**Affiliations:** ^1^Departamento de Microbiología, Escuela Nacional de Ciencias Biológicas, Instituto Politécnico Nacional, Ciudad de México, Mexico; ^2^Departamento de Biomedicina Molecular, Centro de Investigación y de Estudios Avanzados del Instituto Politécnico Nacional, Ciudad de México, Mexico; ^3^Grupo de Biotecnología Molecular, Grupo de Bioinformática y Biología Computacional, Corporación CorpoGen, Bogotá, Colombia; ^4^Facultad de Ingeniería y Ciencias Básicas, Universidad Central, Bogotá, D.C., Colombia; ^5^Unidad de Investigación Médica en Enfermedades Infecciosas y Parasitarias, Hospital de Pediatría, Centro Médico Nacional Siglo XXI, Instituto Mexicano del Seguro Social, Ciudad de México, Mexico; ^6^Departamento de Inmuno-Bioquímica, Instituto Nacional de Perinatología Isidro Espinosa de los Reyes, Ciudad de México, Mexico; ^7^Laboratorio de Bioinformática y Biotecnología Genómica, Departamento de Bioquímica, Escuela Nacional de Ciencias Biológicas, Instituto Politécnico Nacional, Ciudad de México, Mexico; ^8^Departamento de Medicina Preventiva, Facultad de Medicina, Universidad Autónoma de Madrid, Madrid, Spain; ^9^Grupo de Genética de Micobacterias, Universidad de Zaragoza, IIS Aragón, Zaragoza, Spain; ^10^Centro de Investigación Biomédica en Red (CIBER) Enfermedades Respiratorias, Instituto de Salud Carlos III, Madrid, Spain; ^11^Servicio de Microbiología, Hospital Universitario Miguel Servet, Zaragoza, Spain

**Keywords:** *BCG_2177c* gene, TetR family, lipid environment, gene expression, transcriptomics by RNAseq, macrophages response, *Mycobacterium bovis* BCG, *mycobacteria* infection

## Abstract

Mycobacteria, like other microorganisms, survive under different environmental variations by expressing an efficient adaptive response, oriented by regulatory elements, such as transcriptional repressors of the TetR family. These repressors in mycobacteria also appear to be related to cholesterol metabolism. In this study, we have evaluated the effect of a fatty acid (oleic–palmitic–stearic)/cholesterol mixture on some phenotypic and genotypic characteristics of a tetR-mutant strain (*BCG_2177c* mutated gene) of *M. bovis* BCG, a homologous of *Rv2160A* of *M. tuberculosis*. In order to accomplish this, we have analyzed the global gene expression of this strain by RNA-seq and evaluated its neutral-lipid storage capacity and potential to infect macrophages. We have also determined the macrophage response by measuring some pro- and anti-inflammatory cytokine expressions. In comparison with wild-type microorganisms, we showed that the mutation in the *BCG_2177c* gene did not affect the growth of *M. bovis* BCG in the presence of lipids but it probably modified the structure/composition of its cell envelope. Compared to with dextrose, an overexpression of the transcriptome of the wild-type and mutant strains was observed when these mycobacteria were cultured in lipids, mainly at the exponential phase. Twelve putative intracellular redox balance maintenance genes and four others coding for putative transcriptional factors (including WhiB6 and three TetR-like) were the main elements repeatedly overexpressed when cultured in the presence of lipids. These genes belonged to the central part of what we called the “genetic lipid signature” for *M. bovis* BCG. We have also found that all these mycobacteria genotypic changes affected the outcome of BCG-infected macrophages, being the mutant strain most adapted to persist longer inside the host. This high persistence result was also confirmed when mutant-infected macrophages showed overexpression of the anti-inflammatory cytokine TGF-β versus pro-inflammatory cytokines. In summary, the lack of this TetR-like repressor expression, within a lipid environment, may help mycobacteria overcome intracellular redox stress and survive longer inside their host.

## Introduction

Tuberculosis (TB) is a major public health problem worldwide. According to the World Health Organization, in 2020 there were an estimated 9.9 million new TB cases around the world with 1.3 million deaths (WHO, 2021). Additionally, it is assumed that one-quarter of the world’s population is infected with latent *Mycobacterium tuberculosis* (*Mtb*) (LTBI) (Houben and Dodd, 2016). People living with LTBI are asymptomatic and do not transmit the infection but face the possibility of developing active TB (Menzies et al., 2018).

It is considered that *Mtb* establishes long-term LTBI inside the granuloma, surrounded by foamy macrophages and with lipid bodies accumulated in its cytoplasm (Santucci et al., 2016; Prosser et al., 2017). Host-derived lipids (like cholesterol and fatty acids) have been identified as the primary carbon sources for *Mtb* adaptation to the granuloma (Santucci et al., 2016; Aguilar-Ayala et al., 2017; Dong et al., 2021). Also, the caseation of the granuloma correlates with changes in host lipid metabolism (Kim et al., 2010). The relevance of lipids during infection was further demonstrated by Ayyappan et al. (2019) studying a “fatless” model of infection in mice. In their work, mice aerosol infected with *Mtb* showed bacillary counts inside adipose tissue, and those counts increased in the lungs in association with fat cells. More recently, it has been established that alterations in the homeostasis of bone marrow, a high lipid-content tissue, preclude the clinical manifestations of TB (Tamburini et al., 2021). These lipids have also been reported to ameliorate redox stress and help develop drug tolerance during the dormant stage of *Mtb* (Rodríguez et al., 2014; Soto-Ramirez et al., 2017).

The *Mtb* metabolism of cholesterol and fatty acids is controlled by enzymatic reactions that participate in lipid internalization, fatty acid β-oxidation, glyoxylate cycle, and synthesis/storage of triacylglycerols (TAG). It has been observed that a loss in any of these pathways leads to the inability of *Mtb* to establish chronic infection (Fozo and Rucks, 2016; Dong et al., 2021). *Mtb* has an unusual capacity to catabolize cholesterol, an abundant component of host cell membranes (Fine-Coulson et al., 2012). This capacity is used by the pathogen to establish a chronic infection (Pandey and Sassetti, 2008). However, cholesterol catabolism produces the generation of propionyl-CoA that becomes toxic when it accumulates in the cell. This toxicity is ameliorated by generating branched-chain lipids which the mycobacteria use to construct their cell wall (Pandey and Sassetti, 2008; Lovewell et al., 2016).

The genetic regulation of some enzymes involved in mycobacterial lipid metabolism is controlled by regulatory elements named TetR-like transcriptional repressors, such as Mce3R, FdmR, KstR, KstR2, and Fad35R (Kumar et al., 2003; Ouellet et al., 2011; Anand et al., 2012; García-Fernández et al., 2014). The TetR family of transcriptional repressors as regulators is widely distributed among bacteria. They have a characteristic HTH DNA-binding motif, which interacts with the DNA sequence of palindromic promoter regions. The stretch that best defines this profile was analyzed by Ramos et al. (2005), showing a region of 47 amino acid residues found in this motif. The transposon 10 (Tn10) of *Escherichia coli* is the prototype of TetR regulators, which regulates the expression of the tetracycline efflux pump in Gram-negative bacteria (Orth et al., 2000). However, TetR regulators are widely distributed among bacteria and control a broad range of processes, including fatty acid biosynthesis in *Mtb* (Lara et al., 2020).

It is known that TetR functions as a repressor, when an inducer molecule disrupts its binding to DNA and promotes gene transcription through a conformational change in the protein. For KstR and KstR2, the inducer is cholestenone, an intermediate of cholesterol catabolism, whereas palmitoyl-CoA (activated-fatty acid) acts as an inducer of Fad35R, which highlights the critical role of cholesterol and fatty acids as regulatory molecules (Ramos et al., 2005; Bertram and Hillen, 2008; Balhana et al., 2015).

Kendall et al. (2007) have demonstrated that KstR (*Rv3574*) directly controls the expression of 74 genes in *Mtb* that are related to lipid metabolism. These KstR-regulated genes have been induced during the infection of macrophages and shown to be essential for *Mtb* survival in mice (Schnappinger et al., 2003). Additionally, while KstR can repress the *igr* operon genes, which are involved in the degradation of the aliphatic chain of cholesterol (Chang et al., 2009), KstR2 regulates the expression of 15 genes involved in cholesterol catabolism and in the β-oxidation of fatty acids (Kendall et al., 2010; Ouellet et al., 2011; García-Fernández et al., 2014). Fad35R, another TetR repressor, regulates the catabolism of fatty acids in *Mtb* and can sense the levels of active fatty acids altering its DNA-binding activity, controlling in this way the expression of its downstream genes in a metabolite-dependent manner (Anand et al., 2012).

In a previous work carried out by our group, a mutant strain of *M. bovis* BCG (BCG_2177cTngfp) was selected (using GFP fluorescence) from a mutant library generated by transposition. This mutant showed the highest level of long-standing fluorescence at the stationary phase. A few interesting morphological and physiological changes were also observed when this strain was cultured in the presence of cholesterol, i.e., the mutant strain seemed to accumulate intracellular neutral lipids and they were arranged in a microscopic cord-like structure (Otal et al., 2017). These results guided us toward using BCG_2177cTngfp as a suitable strain for studying the role of the *BCG_2177c* gene (a homolog of *Rv2160A* of *Mtb*) in this slow-growing mycobacterium, which encodes for a TetR-like repressor during lipid metabolism (fatty acids/cholesterol mixture).

Therefore, the aim of our work here was to determine the global effect of a mixture of fatty acids–cholesterol on some phenotypic and genotypic characteristics of a TetR-like repressor mutant strain of *M. bovis* BCG, which could be extrapolated to other members of the *Mtb* complex (MTBC). We have found that the particular mutation of the *BCG_2177c* gene, together with the presence of a lipid mixture in the culture, did not affect the cell growth of *M. bovis* BCG but stimulated the overexpression of 20 genes and allowed this mycobacterium to persist longer inside macrophages. We have also shown that intracellular neutral-lipid storage increased in both wild-type and mutant strains cultured in a lipid mixture at the stationary phase. A loss of acid-fastness was also found in the mutant strain under these conditions. We propose that together with the BCG-infected macrophage results, these changes may aid mycobacteria to evade oxidative mechanisms of the innate immune response and lead them to modulate the host cytokine response during infection.

## Materials and Methods

### Bacterial Strain and Culture Conditions

*M. bovis* BCG Pasteur 1173P2 (wtBCG) and *M. bovis* BCG *2177c_Tngfp* (mtBCG), a mutant strain obtained by transposition of *Tngfp* in the *BCG_2177c* gene (TetR repressor) (Otal et al., 2017), were grown with agitation at 37°C in Middlebrook 7H9 broth containing 0.2% dextrose (control medium) or in a lipid mixture of oleic acid, palmitic acid, and stearic acid at a final concentration of 0.001% each, plus 0.01% cholesterol, prepared as a 5% stock solution of cholesterol dissolved in a 1:1 mixture of ethanol/tyloxapol, as previously reported (Soto-Ramirez et al., 2017). No Tween 80 was added to the 7H9 medium. In this way, we analyzed four conditions for each strain: exponential and stationary phases and the presence of lipids or dextrose as carbon sources. In order to obtain cultures at the exponential and stationary phases, mycobacterial growth was monitored by measuring OD at 600 nm and CFU/mL every 24 h for 30 days. Cultures from exponential phases were therefore collected at day 5 from lipids, and at day 8 from dextrose, while all stationary phase cultures were collected at day 25 regardless of the main carbon source. Cells from each condition were harvested for microscopy analyses, for RNA isolation, and for the THP-1 macrophage infections.

### RNA Isolation

Total RNA from each culture condition was isolated as previously reported (Soto-Ramirez et al., 2017) and purified using TRIzol™ Reagent (Invitrogen, Carlsbad, CA, USA). As two strains (wtBCG and mtBCG) were used and four conditions were tested (two growth phases: exponential and stationary, and two main carbon sources: dextrose and lipids), eight RNA samples were acquired. Finally, a total of 16 RNA samples were obtained because two biological replicates were examined. All of them achieved the RNA quality required for RNA-seq analysis: 1) RNA concentration from 0.4 to 1.6 µg/µL, which was evaluated using Qubit™ RNA HS Assay Kit in the Qubit™ 2.0 fluorometer, 2) DNA absence which was analyzed by qPCR and was not detected, and 3) RNA integrity number (RIN), which was analyzed with a bioanalyzer (Agilent Technologies, Santa Clara, CA, USA) and found to be between 7.0 and 8.5.

### cDNA Library Construction

RNA-seq libraries were prepared in accordance with the previously reported protocols (Waldbauer et al., 2012; Rodríguez et al., 2014). In summary, RNA samples were diluted at a concentration of 0.4 µg/µL for rRNA removal using the MICROBExpress*™* mRNA Enrichment Kit (Invitrogen, USA); mRNA was precipitated and solubilized in 20 µL DEPC-treated water. Purified mRNA was fragmented by divalent-cation hydrolysis (Fragmentation Buffer; Ambion, Austin, TX) at 70°C for 12 min to yield fragment sizes between 60 and 200 nt. After purification using the RNA Clean & Concentrator™ Kit (Zymo Research, Irvine, CA, USA), mRNA samples were subjected to poly(A) tailing and end repairing (NEB Reagents, Ipswich, MA, USA). RNA was treated with Antarctic Phosphatase (New England Biolabs) and then phosphorylated at the 5′ ends with T4 PNK (New England Biolabs). The transcribed strand was labeled by ligation of a 5′ hybrid DNA-RNA primer (5-hybrid-0), and after purification with RNAClean XP beads (Beckman Coulter Genomics, Brea, CA, USA), the first-strand cDNA synthesis reaction was carried out with SuperScript II Reverse Transcriptase (Invitrogen, USA) and Illumina’s poly(T) primer (IGA-dT16-VN). The reaction components of cDNA synthesis were removed by treatment with Agencourt AMPure XP SPRI beads (Beckman, USA), and primary transcripts were enriched with a high-fidelity polymerase (ACCUZYME™ Kit, Bioline, London, UK) and the Illumina spacers as primers. Illumina adaptors and bar codes were ligated by PCR in accordance with the manufacturer’s instructions. Libraries were purified with SPRI beads (Beckman) and evaluated by Qubit™ 2.0 and Bioanalyzer (Agilent Technologies). Finally, we obtained cDNA libraries with a size of 350 bp and with concentrations from 12.5 to 35.7 ng/µL. Libraries were sequenced at Macrogen company (Seoul, Korea) using the HiSeq 2500 platform of Illumina (San Diego, CA, USA).

### RNA-Seq Data Analysis

RNA-seq data were obtained *via* HiSeq 2500 System using HCS v2.2 software, and the reads obtained were processed to remove poly(A) and spacer sequences, and only those with quality scores of Q30 and a minimal length of 50 bases were used for analysis. Assessment of read quality was carried out using the FASTX-Toolkit v. 0.0.13 (http://hannonlab.cshl.edu/fastx_toolkit/). Reads were cleaned *in silico* from rRNA sequences in order to increase the efficiency of the assembly process using the SortMeRNA software as previously described (Kopylova et al., 2012). rRNA-free mRNA were mapped against the *M. bovis* BCG-Pasteur reference genome (accession no. **NC_008769.1**) with Bowtie v.1.1.2 (http://bowtie-bio.sourceforge.net/index.shtml) and visualized with the Artemis sequence visualization and annotation tool v 16.0 (http://www.sanger.ac.uk/science/tools/artemis). Reads that were mapped to more than one site were excluded. For the analysis, only those reads for which 50% of the sequence length fell within the annotated ORF were considered to be a part of the ORF. At the end, an average of 5.8 million of reads per library were obtained. The coverage estimation of the transcriptome of *M. bovis* BCG was determined by saturation curves from the “**counts**” values (number of reads that have been assigned to a gene) determined using the featureCounts software v 1. 5. 0 (http://subread.sourceforge.net). In order to know the differential genetic expression of strains in the studied culture conditions, the initial relation of the expression in both biological replicates for each condition was achieved by calculating the Euclidean distance: this considers the variance of the normalized reading counts (expression values adjusted to the total readings in each sample) of each library to construct a distance matrix, using the DESeq package version 1.22.1 (http://bioconductor.org/packages/release/bioc/html/DESeq.html). Differential gene expression analysis based on the negative binomial distribution (DESeq) was used in order to compare genetic expressions among all conditions. This package allowed us to know ***log2 fold-change values*** from the counts of each gene comparing two conditions considering p < 0.05% (which controls the false discovery rate, FDR). Overexpressed genes (p < 0.05) of both strains in the presence of a lipid mixture were analyzed, and 10 most representative genes of this comparison were validated by RT-qPCR (**Table S1**). An *M. bovis* BCG reference genome (NC_008769.1) was annotated using the protocol of “*Rapid Annotation using Subsystem Technology*” (RAST, v2.0, http://rast.nmpdr.org). This process grouped *M. bovis* BCG genes in 27 functional categories according to the prokaryote genomes deposited in this database.

### Microscopy Assays

Microscopic differences and lipid storage were evaluated by Auramine-Nile Red stain. Smears of *M. bovis* BCG (wtBCG and mtBCG) from each condition were stained with Auramine O (TB Fluorescent Stain Kit M, Becton Dickinson, Sparks, MD) and with Nile Red (Invitrogen/Molecular Probes, Carlsbad, CA) according to the manufacturers’ instructions. Samples were protected with a coverslip using VECTASHIELD™ as a mountain medium and examined by confocal laser microscopy (LSM Pascal, Carl Zeiss, Oberkochen, Germany). Images were analyzed by the LSM 5 image browser (https://www.embl.de/eamnet/html/body_image_browser.html).

### Cell Line Culture and Infection

Human monocytes THP1 (ATCC TIB-202) were cultured in RPMI 1640 medium (ATCC^®^ 30-2001, Manassas VA, USA) supplemented with 10% heat-inactivated fetal bovine serum (FBS) and 0.05 mM of β-mercaptoethanol and incubated at 37°C in a 5% CO_2_ incubator until a density of 1 × 10^6^ cells/mL was reached. Cells were counted in a Neubauer chamber after trypan blue staining (0.2%). THP1 monocytes were differentiated to macrophages using phorbol-12-myristate-13-acetate (PMA, Sigma-Aldrich, St. Louis, MO, USA) at a concentration of 10 nM for 72 h. Both strains of BCG from each condition were added to the macrophage culture at a multiplicity of infection (MOI) of 5 (5:1). Infected cells were incubated during 4 h, then washed with PBS in order to eliminate extracellular mycobacteria, as previously reported (Helguera-Repetto et al., 2014). This *momentum* was considered as time 0 h for the infection kinetic. Finally, infected cells were incubated in RPMI medium supplemented with 3% FBS.

### Macrophage Monolayer Integrity Kinetics

Kinetics were made in eight-well slides (Nunc, Thermo Scientific, Rockford, IL, USA) plated with a MOI of 5. Macrophage infection was evaluated each 24 h during a 96-hpi kinetic. At each time (0, 24, 48, 72, and 96 hpi), cells were fixed with 4% paraformaldehyde (PFA), stained using the Kinyoun staining method, and analyzed by optical microscopy (1000× Carl Zeiss Axiostar Plus microscope, Dresden, Germany). The percentage of integrity and the percentage of infection were determined by counting macrophages in infected and non-infected cultures (control) at each time. One hundred percent of integrity corresponded to the macrophage’s total number found in non-infected cultures. All experiments were performed in triplicate.

### Macrophage–Mycobacteria Interaction Kinetics

Kinetics were evaluated in 24-well plates (Costar, Sigma-Aldrich) with 5 × 10^5^ macrophages/well, from 0 to 96 hpi. Once each time was reached, 500 µL of supernatant from each well was collected, centrifuged, and stored at -70°C for interleukin level determination (see below). Five hundred microliters of 0.05% Tween 80 (Sigma-Aldrich, USA) were added to the remaining cells in order to lyse the macrophage monolayer, and the viable mycobacterial counts (CFU/mL) in 7H10 medium (Becton Dickinson, MD, USA) were carried out each time; colonies were counted after 7 to 10 days of growth on agar. These CFUs/mL were compared with those found with each microorganism culture in RPMI medium without macrophages. All experiments were performed in triplicate.

### Production of Reactive Oxygen Species by Macrophages

Macrophages (1.5 × 10^5^) were plated in each 96-well flat-bottom plate (Costar, Sigma-Aldrich) and infected with each mycobacterial suspension from each environmental condition (MOI 5:1). From 0 to 96 hpi, 100 µL of supernatant was collected for lactate dehydrogenase (LDH) quantification. Twenty microliters of 0.1% NBT (Sigma-Aldrich) was added to the remaining volume of each well, and plates were incubated at 37°C for 15 min in darkness. The reaction was stopped by adding 30 µL of 1% SDS/0.8M NaOH solution, and plates were incubated overnight in darkness in order to solubilize formazan precipitates. Color intensity was quantified with a microplate reader (Thermo Multiskan EX, Waltham, MA, USA) at 570 nm. A basal control for ROS production was included, which consisted of 20 nM PMA-stimulated macrophages without mycobacteria. A positive control for ROS production (100 nM PMA-stimulated macrophages) was also included.

### Cytotoxic Effect of THP1 Macrophages

This parameter was evaluated by measuring LDH activity released from the damaged cells, using the Cytotoxicity Detection Kit^PLUS^ LDH (Roche, Mannheim, Germany) in a 96-well assay with 100 µL of supernatant of each infection. The color intensity was measured at 490 nm in a microplate reader (Thermo Multiskan EX, USA).

For this cytotoxic effect, three experimental controls were used in order to estimate the percentage of cytotoxicity: 1) background control (culture medium), 2) low control (macrophage monolayer without mycobacteria), and 3) high control (macrophage monolayer treated with the Lysis Reagent, included in the kit). The values of absorbance obtained from each sample and the high LDH control were normalized by subtracting the background of the culture medium from the absorbance obtained in the low control. Then, the percentage of cytotoxicity was calculated considering 100% of cytotoxicity as the one obtained in the high control.

### Production of Pro-Inflammatory and Anti-Inflammatory Cytokines by Macrophages

Fifty microliters of each supernatant from the interaction kinetics was used to determine the level of some pro-inflammatory (IL-1β, IL-8, and TNF-α) and anti-inflammatory (IL-10 and TGF-β) cytokines by ELISA (Single-Analyte ELISArray, Qiagen, Germantown, MD, USA) according to the manufacturer’s protocol (Single-Analyte ELISArray Handbook, Qiagen; https://www.qiagen.com).

### Statistical Analysis

Except for the RNA-seq analysis, at least three independent determinations (biological replicates) were carried out in triplicate (n = 9). GraphPad Software v 6.0 (La Jolla CA, USA) was used for statistical analysis. Multiple comparisons were achieved using two-way or three-way ANOVA and the Dunnett’s or Tukey’s posttest, respectively; in both, test p-values ≤ 0.05 were considered significant.

## Results

In order to further understand the response of mycobacteria to a lipid environment, which is thought to be involved throughout a host’s infection, we decided to analyze the *in vitro* effect of a fatty acid/cholesterol mixture on the global expression and on several phenotypic characteristics of an *M. bovis* BCG strain, previously mutated (mtBCG) in the *tetR*-gene (*BCG_2177c*) by our working group (Otal et al., 2017).

As a first step, mutant and wild-type strains were cultured in a lipid mixture (fatty acids and cholesterol) or dextrose, and growth curves were obtained (**Figure S1**). According to the exponential-phase slopes, both strains cultured in lipids showed higher growth rates than those found in dextrose, regardless of their genetic background. However, at the late stationary phase (day 30), the final cell mass reached was not related to the carbon source (**Figure S1**).

Total RNA was isolated from all culture conditions and analyzed by RNA-seq. A plateau reached by saturation curves from sequences of all cDNA libraries showed an adequate coverage of the entire genome of *M. bovis* BCG (***NC_008769.1***) (**Figure S2**). All sequences obtained from RNA-seq experiments are reported in the NCBI Gene Expression Omnibus (Edgar et al., 2002) under access number GSE175579.

### Differential Gene Expression by Functional Category

**Figure 1:**
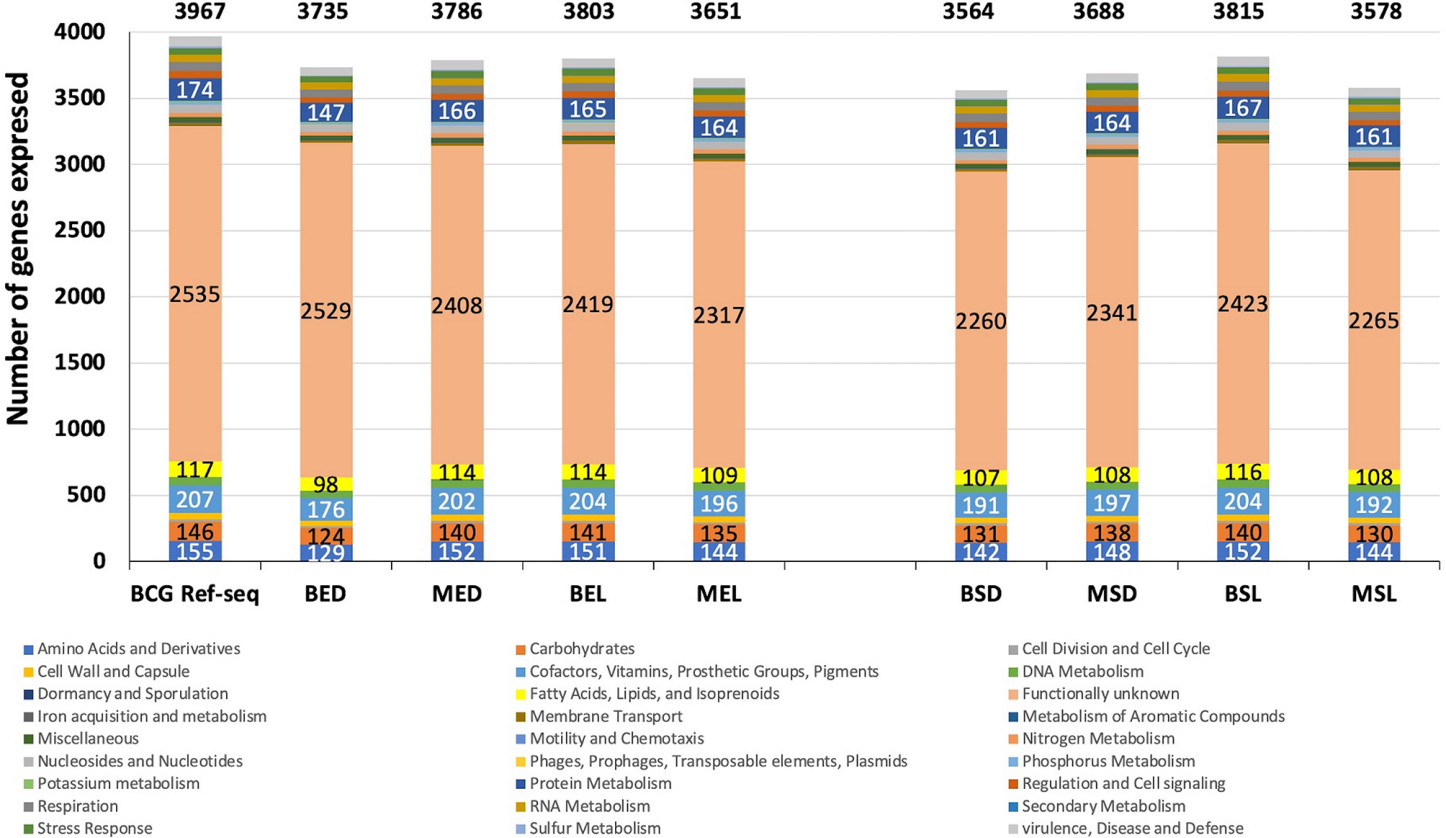
Global gene expression of wtBCG and mtBCG under different environmental conditions. Gene clustering by functional categories was carried out using the RAST server (http://rast.theseed.org/FIG/rast.cgi) and the reference genome of *M. bovis* BCG available in the NCBI (***NC_008769***). Six functional categories, (**1)** functionally unknown ORF; **2)** cofactors, vitamins, and prosthetic groups; **3)** protein metabolism; **4)** amino acids and derivatives; **5)** carbohydrates; and **6)** fatty acids, lipids, and isoprenoids (in almost all cultured conditions), showed changes in the expression of total *M. bovis* BCG genes.

To facilitate the gene expression comparison in each studied condition for wtBCG and mtBCG strains, the expression of the total number of genes (by functional category) was determined. Using the RAST server, all genes reported in the *M. bovis* BCG reference genome (***NC_008769.1***) were classified into 27 categories (**Figure 1**, BCG-Ref-seq bar). This genome was also used as a template in order to sort out all expressed BCG genes in each library (**Figure 1**).

During the exponential and stationary phases of growth, the expression of the highest number of genes was found in the wild-type strain cultured in lipids (3,803 for BEL and 3,815 for BSL). The majority of these genes corresponded to **1)** functionally unknown ORF (2,419 and 2,423 genes, respectively); **2)** cofactors, vitamins, and prosthetic groups (204 genes); **3)** protein metabolism (165 and 167 genes, respectively); **4)** amino acids and derivatives (151 and 152 genes, respectively); **5)** carbohydrates (141 and 140 genes, respectively); and **6)** fatty acids, lipids, and isoprenoids (114 and 116 genes, respectively). On the other hand, the lowest number of expressed genes (3,564) occurred in the wild-type strain cultivated in the presence of dextrose (BSD) (**Figure 1** and **Table S2**).

### Expression Rates of *M. bovis* BCG Genes

**Figure 2:**
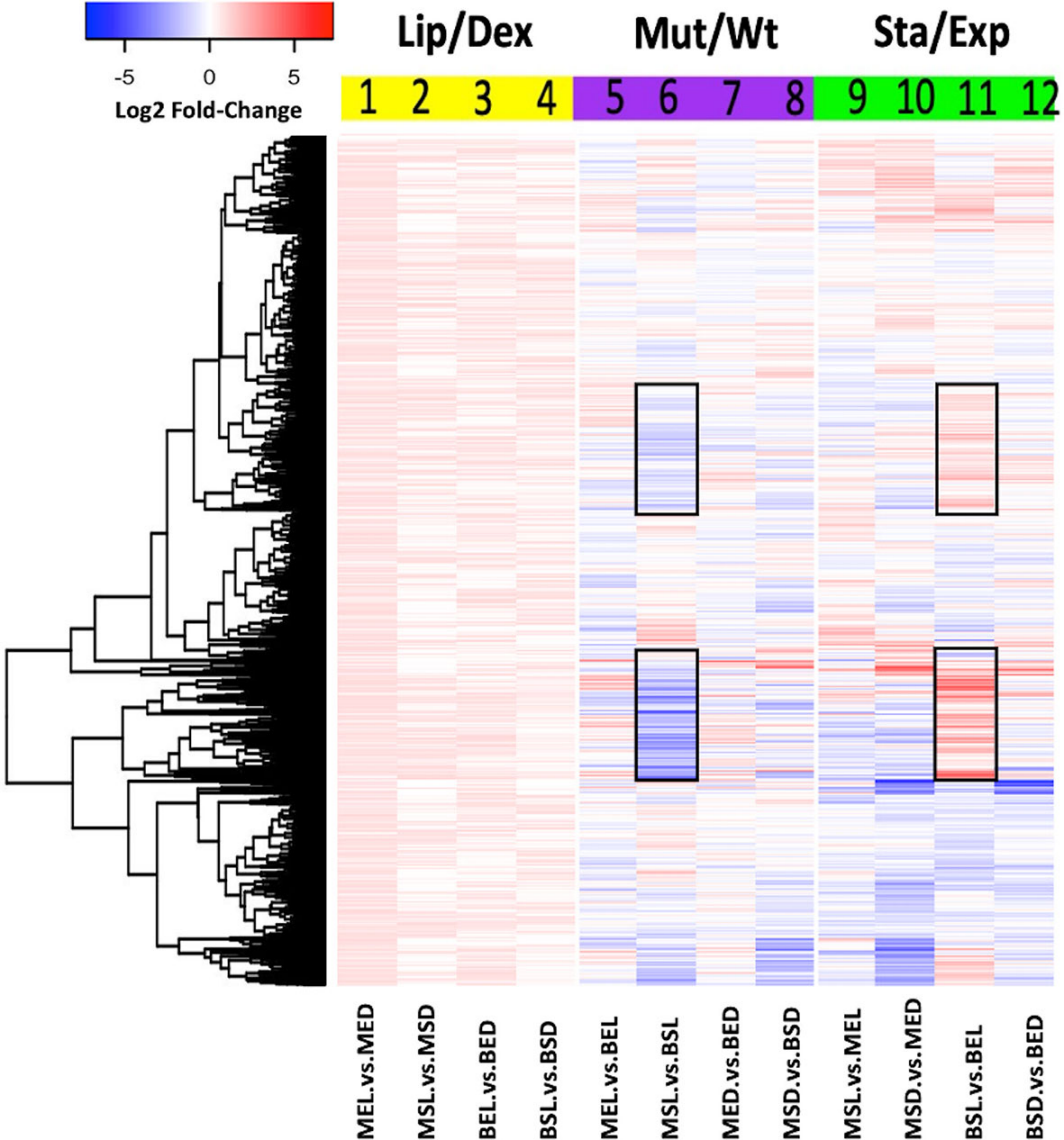
Global transcriptomic expression of *M. bovis* BCG genes. Heat map showed the *log2 fold-change* values of each gene expression under three different analyses: lipid effect (yellow block), *BCG_2177c* mutation effect (purple block), and growth phase effect (green block). All experiments were performed as previously described in the methodology section. wtBCG or mtBCG strains cultured in dextrose or lipids during the exponential (BED, wtBCG dextrose; BEL, wtBCG lipids; MED, mtBCG dextrose; MEL, mtBCG lipids) or stationary phase (BSD, wtBCG dextrose; BSL, wtBCG lipids; MSD, mtBCG dextrose; MSL, mtBCG lipids).

**Figure 3:**
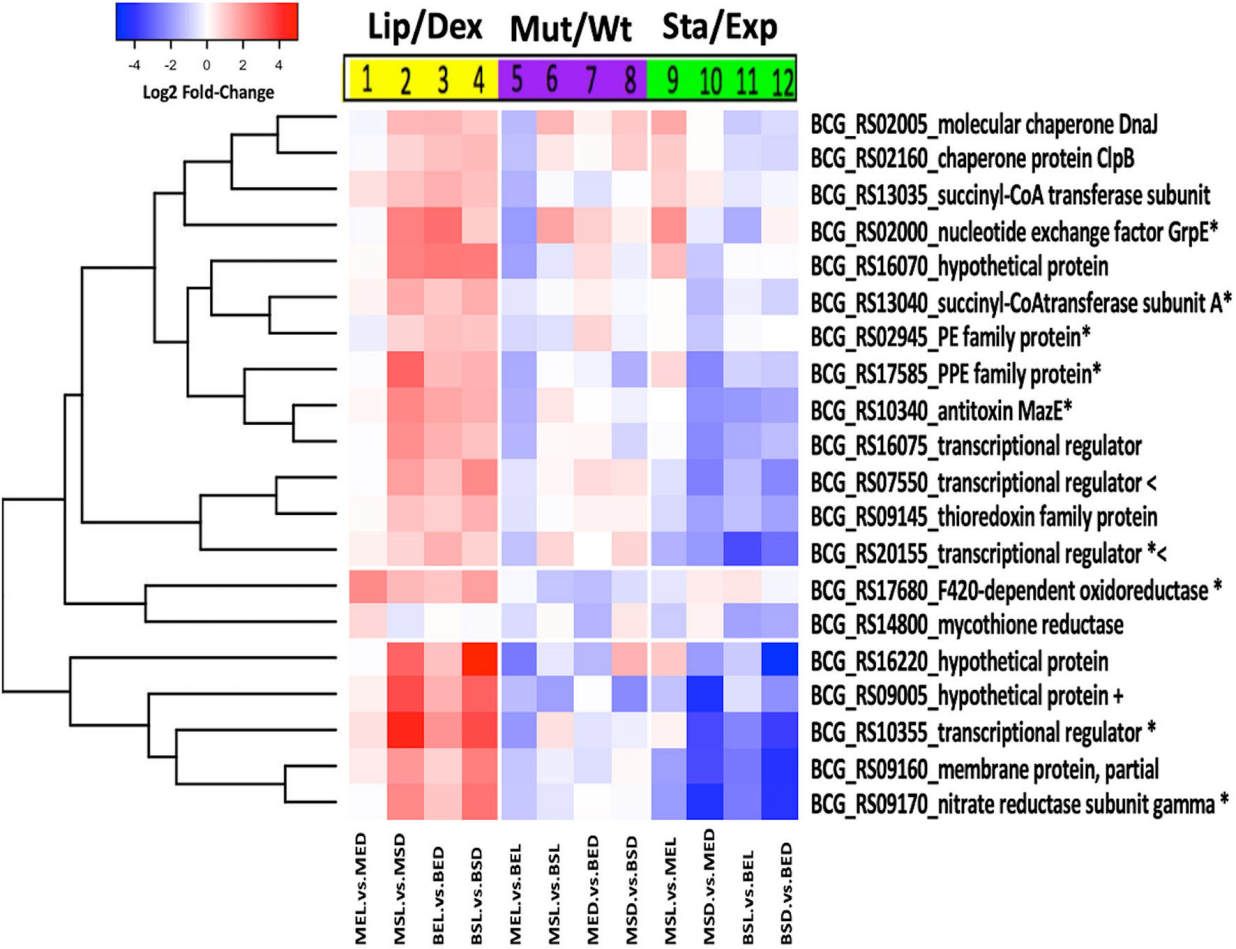
Expression patterns of *M. bovis* BCG lipid signature genes. *Log2 fold-change* values of each of the 20 genes of the lipid signature for *M. bovis* BCG are observed under three different analyses: lipid effect (yellow block), *BCG_2177c* mutation effect (purple block), and growth phase effect (green block). All experiments were performed as previously described in the methodology section. wtBCG or mtBCG strains cultured in dextrose or lipids during exponential (BED, wtBCG dextrose; BEL, wtBCG lipids; MED, mtBCG dextrose; MEL, mtBCG lipids) or stationary phases (BSD, wtBCG dextrose; BSL, wtBCG lipids; MSD, mtBCG dextrose; MSL, mtBCG lipids).

**Figure 4:**
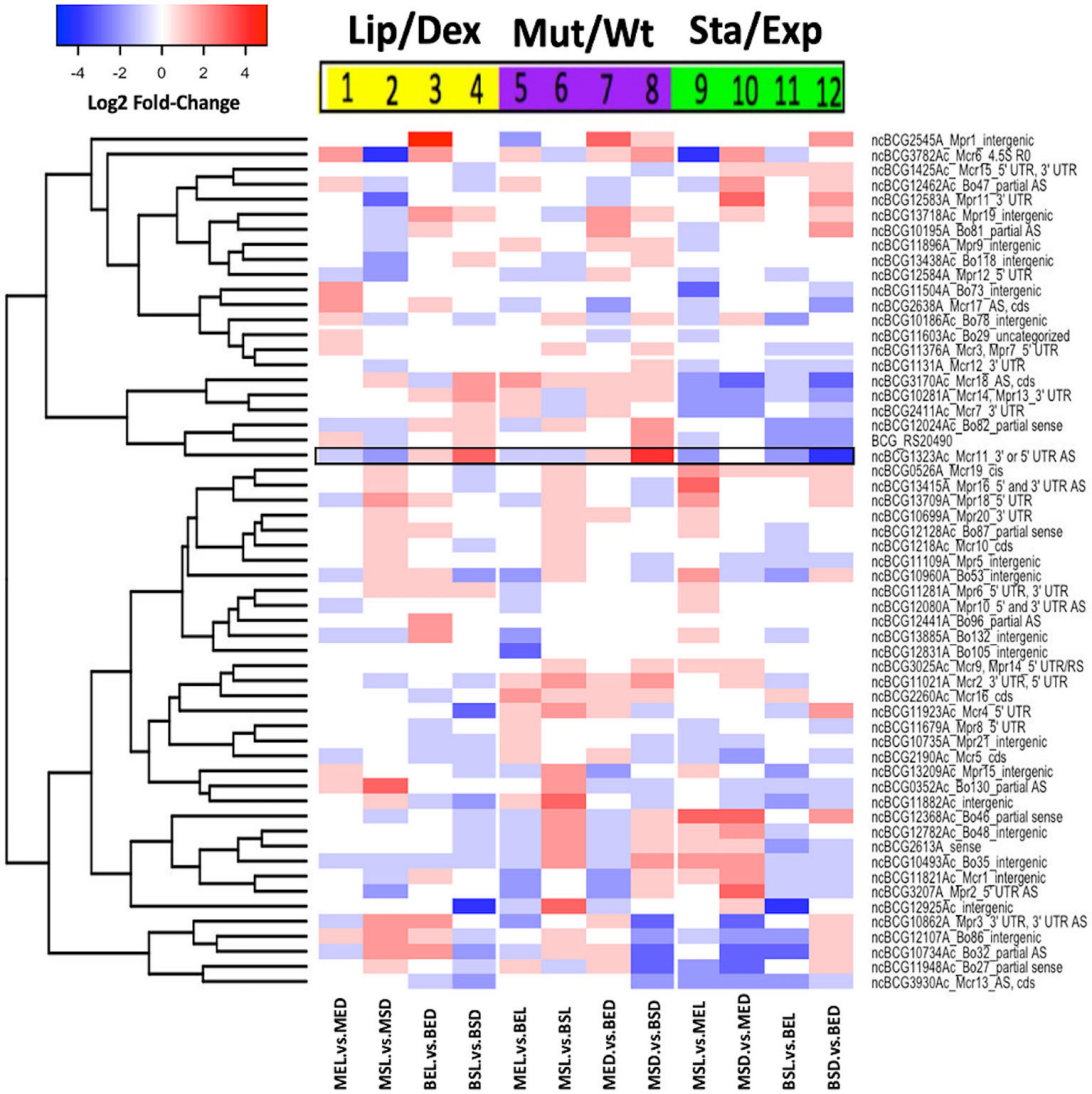
Comparative expression of non-coding RNAs from *M. bovis* BCG. Log2 fold-change values of ncRNAs are shown. All experiments were performed as previously described in the methodology section. wtBCG or mtBCG strains cultured in dextrose or lipids during exponential (BED, wtBCG dextrose; BEL, wtBCG lipids; MED, mtBCG dextrose; MEL, mtBCG lipids) or stationary phases (BSD, wtBCG dextrose; BSL, wtBCG lipids; MSD, mtBCG dextrose; MSL, mtBCG lipids).

In order to determine the global changes in BCG gene expression, all data from each library were analyzed using the DESeq package. Three comparisons were evaluated according to the variables used for this study (**Figures 2**–**4**): **1)** the carbon source (dextrose or lipids, yellow blocks), **2)** the BCG strain (wild-type or mutant, purple blocks), and **3)** the gene expression in each growth phase (exponential or stationary phase, green blocks). In all comparisons, heat maps generated from the log2 fold-expression change values were obtained. An increase in the global expression of genes in both strains was observed when mycobacteria were grown in the presence of lipids (**Figure 2**, yellow block). Here, the exponential-phase mutant strain (MEL) had the highest global overexpression rates of transcripts (**Figure 2**, lane 1). When the effect of the mutation was evaluated (**Figure 2**, purple block), the stationary-phase mutant strain (MSL) grown in lipids yielded the largest number of low-expressed transcripts, in comparison with the wild-type strain (**Figure 2** lane 6).

Some of these genes include *BCG_RS05655* (ESAT-6-like protein EsxL, *Rv1037c*), *BCG_RS02210* (hypothetical protein, *Rv0395*), *BCG_RS00345* (acyl carrier protein, *Rv0033*), *BCG_RS01320* (probable conserved transmembrane protein, *Rv0219*), *BCG_RS02880* (methyltransferase domain of sarcosine-dimethylglycine methyltransferase, *Rv1523*), *BCG_RS19930* (putative uncharacterized protein BCG_3881, *Rv3819*), *BCG_RS11675* (malonyl CoA-acyl carrier protein transacylase, *Rv2243*), and *BCG_RS03640* (DNA-binding protein, CopG family, *Rv0662c*) (**Figure 2** lane 6).

To facilitate the interpretation of the biological effect of the three variable conditions mentioned above (carbon source/growth phase/*tet*R mutation), we selected 19 genes, which under most conditions were found to be overexpressed in both strains in the presence of lipids (with p < 0.05). These genes encoded proteins that participate as BCG transcriptional regulators [*BCG_RS10355* (*Rv1994c*), *BCG_RS16075* (*Rv3095*), *BCG_RS07550* (*Rv1395*), and *BCG_RS20155* (*Rv3862c*)], as PE and PPE families [*BCG_RS02945* (*Rv0532*) and *BCG_RS17585* (*Rv3350c*), respectively], as chaperones [*BCG_RS02160* (*Rv0384c*) and *BCG*_*RS02005* (*Rv0352*)], as toxin–antitoxin system (*BCG_RS10340*), as nucleotide exchange factors and enzymatic subunits [*BCG_RS02000* (*Rv0351*), *BCG_RS09170, BCG_RS13035* (*Rv2503c*) and *BCG_RS13040* (*Rv2504c*), respectively], as thioredoxin protein [*BCG_RS09145* (*Rv1732c*)], as membrane protein [*BCG_RS09160* (*Rv1735c*)], as POX class F420-dependent oxidoreductase [*BCG_RS17680* (*Rv3369*)], and as those whose function is not known (hypothetical) [*BCG_RS09005* (*Rv1706c*), *BCG_RS16220* (*Rv3126c*), and *BCG_RS16070* (*Rv3094c*)] (**Table 1**). Another gene (upregulated only in the mutant strain at its exponential phase in the presence of lipids) was also selected [*BCG_RS14800* (*Rv2855*)] (**Table 1**). These genes were named as **“the lipid signature of *M. bovis* BCG”** considering their significant gene overexpression in the presence of lipids. The differential expression of these 20 genes was validated by analyzing the expression of 50% of them by qRT-PCR in all conditions. A correlation with the changes identified by RNA-seq was found in all cases (**Figure S3**).

**Table 1:** Log2 Fold-Change and p-values of *M. bovis* BCG lipid signature genes.

Gen_ID and function	*M. tuberculosis gene Id (Rv)*	log2 fold change				FDR (p < 0.05)			
		MEL vs. MED	MSL vs. MSD	BEL vs. BED	BSL vs. BSD	MEL vs. MED	MSL vs. MSD	BEL vs. BED	BSL vs. BSD
**BCG_RS10355_ ArsR-transcriptional regulator**	*Rv1994c (cmtR)*	0.5715	4.219	1.9932	3.3419	2.86E-01	4.91E-18	1.51E-06	1.66E-10
BCG_RS16075_ transcriptional regulator	*Rv3095*	-0.0387	2.0445	1.4651	1.1437	8.09E-01	1.32E-05	1.32E-04	1.55E-02
BCG_RS07550_ transcriptional regulator	*Rv1395*	-0.0193	1.7685	1.1529	2.1619	9.99E-01	7.78E-04	1.55E-02	2.26E-04
**BCG_RS20155_ WhiB-transcriptional regulator**	*Rv3862c (WhiB6)*	0.2843	0.7769	1.4486	0.831	2.80E-01	6.14E-03	1.44E-05	2.25E-02
**BCG_RS09005_ hypothetical protein**	*Rv1706c*	0.2706	3.3155	1.4703	2.9247	6.84E-01	9.27E-03	3.25E-02	1.05E-03
BCG_RS16220_ hypothetical protein	*Rv3126c*	-0.0211	2.8646	1.1608	4.7296	9.38E-01	1.79E-05	2.52E-02	5.70E-09
BCG_RS16070_ hypothetical protein	*Rv3094c*	0.0896	2.2949	2.4779	2.4281	6.46E-01	8.41E-04	2.52E-04	3.84E-04
BCG_RS09160_hypothetical protein	*Rv1735c*	0.3787	1.9195	0.8584	2.3829	1.35E-01	2.43E-11	6.54E-03	7.43E-13
**BCG_RS10340_antitoxin MazE**	NA	0.1735	2.209	1.6591	1.4597	9.60E-01	1.44E-04	1.45E-04	1.03E-02
BCG_RS02160_chaperone protein ClpB	*Rv0384c*	-0.1056	0.7989	1.1572	1.2688	7.96E-01	2.15E-02	4.75E-04	3.58E-03
BCG_RS02005_ chaperone protein DnaJ	*Rv0352*	-0.1998	1.3411	1.3447	1.0287	8.86E-01	1.12E-03	1.97E-03	4.03E-02
**BCG_RS09170_nitrate reductase subunit gamma**	NA	-0.0222	2.1899	1.0715	2.5803	8.84E-01	3.73E-08	5.90E-03	1.59E-09
**BCG_RS02000_ nucleotide exchange factor GrpE**	*Rv0351 (Hsp70)*	-0.0867	2.3323	2.6738	0.9397	8.27E-01	2.23E-10	1.14E-13	3.13E-02
**BCG_RS02945_PE-PGRS family protein**	*Rv0532 (PE_PGRS6)*	-0.3118	0.7796	1.1844	1.0794	1.83E-01	2.91E-02	2.03E-02	1.91E-02
**BCG_RS17585_PPE Family protein**	*Rv3350c (PPE56)*	-0.0585	2.8694	1.2599	1.4363	7.19E-01	1.38E-07	6.31E-04	1.10E-02
**BCG_RS13040_ succinyl-CoA–3-ketoacid CoA transferase subunit A**	*Rv2504c (ScoA)*	0.2248	1.5409	1.0296	1.5298	5.84E-01	9.36E-05	3.05E-02	9.74E-04
BCG_RS13035_ succinyl-CoA–3-ketoacid CoA transferase subunit B	*Rv2503c (ScoB)*	0.5774	1.0946	1.4432	1.1742	4.38E-01	1.38E-02	1.23E-03	1.93E-02
BCG_RS09145_ thioredoxin protein	*Rv1732c*	0.0948	1.1348	0.8929	1.445	7.39E-01	1.34E-03	2.67E-02	6.89E-04
BCG_RS17680_ PPOX class F420-dependent enzyme*	*Rv3369*	2.149	1.3103	1.0717	1.7568	2.91E-02	1.42E-01	7.36E-02	3.00E-02
BCG_RS14800_mycothione reductase *	*Rv2855 (Mtr)*	0.7303	-0.4723	0.0538	-0.0972	4.19E-02	1.53E-01	2.27E-01	7.05E-01

*Upregulated at the exponential phase in the mutant strain; Genes in bold, their expression was validated by RT-qPCR. Corresponding Rv names were obtained from the KEGG database https://www.genome.jp/kegg/kegg2.html. NA, no-orthologs found in the M. tuberculosis H37Rv genome.

The expression of the “lipid signature of *M. bovis*” genes was further analyzed using three different correlations (lipids/dextrose, mutant/wild-type, and stationary/exponential) with both strains and construction of a new heat map (**Figure 3**). A significant decrease in the expression of all these genes was found in the mutant strain compared to the expression in the wild-type strain during the exponential phase (MEL vs. BEL) (**Figure 3**, lane 5). It was also observed that seven of these genes were overexpressed when the mutant strain was at the stationary phase (**Figure 3**, lane 9) in the presence of lipids. During this growth phase, a significant decrease in the expression of these 20 genes was found when other combinations of carbon sources or types of strains were used (**Figure 3**, lanes 10–12), suggesting that the inhibition of the putative *tetR* gene in the mutant strain, together with lipids, may have an important role in the overexpression of those seven lipid signature genes mentioned above.

### Non-Coding RNA Expression

As the *M. bovis* BCG Pasteur genome has 64 non-coding RNAs (ncRNA) and their expression is known to vary depending on growing conditions (Haning et al., 2014), we decided to construct a heat expression map of ncRNAs only. In this work, at least 50% of the ncRNAs were overexpressed in the mutant strain grown in the presence of lipids (MSL), at the stationary phase of growth (**Figure 4**, lanes 2, 6, and 9). In particular, the *ncBCG1323Ac* (black framed data, **Figure 4**) gene was overexpressed in the wild-type strain grown in lipids (BSL) compared to the same strain cultured in dextrose (BSD) (**Figure 4**, black framed data, lane 4). An overexpression of this same ncRNA was also found in the mutant (MSD) strain in the presence of dextrose compared to the wild-type strain (**Figure 4**, black lane data, lane 8). We also found that the wild-type strain grown in lipids at its stationary phase (BSL) showed the lowest level of ncRNA expression (**Figure 4**, lane 11). We could not detect the expression of eight ncRNA genes in any studied condition (*ncBCG_11448Ac*, *ncBCG_1115Ac*, *ncBCG_s24A*, *ncBCG_2654Ac*, *ncBCG_12882A*, *ncBCG_13661A*, *ncBCG_13719A*, and *13719B*).

### Microscopy Assays

**Figure 5:**
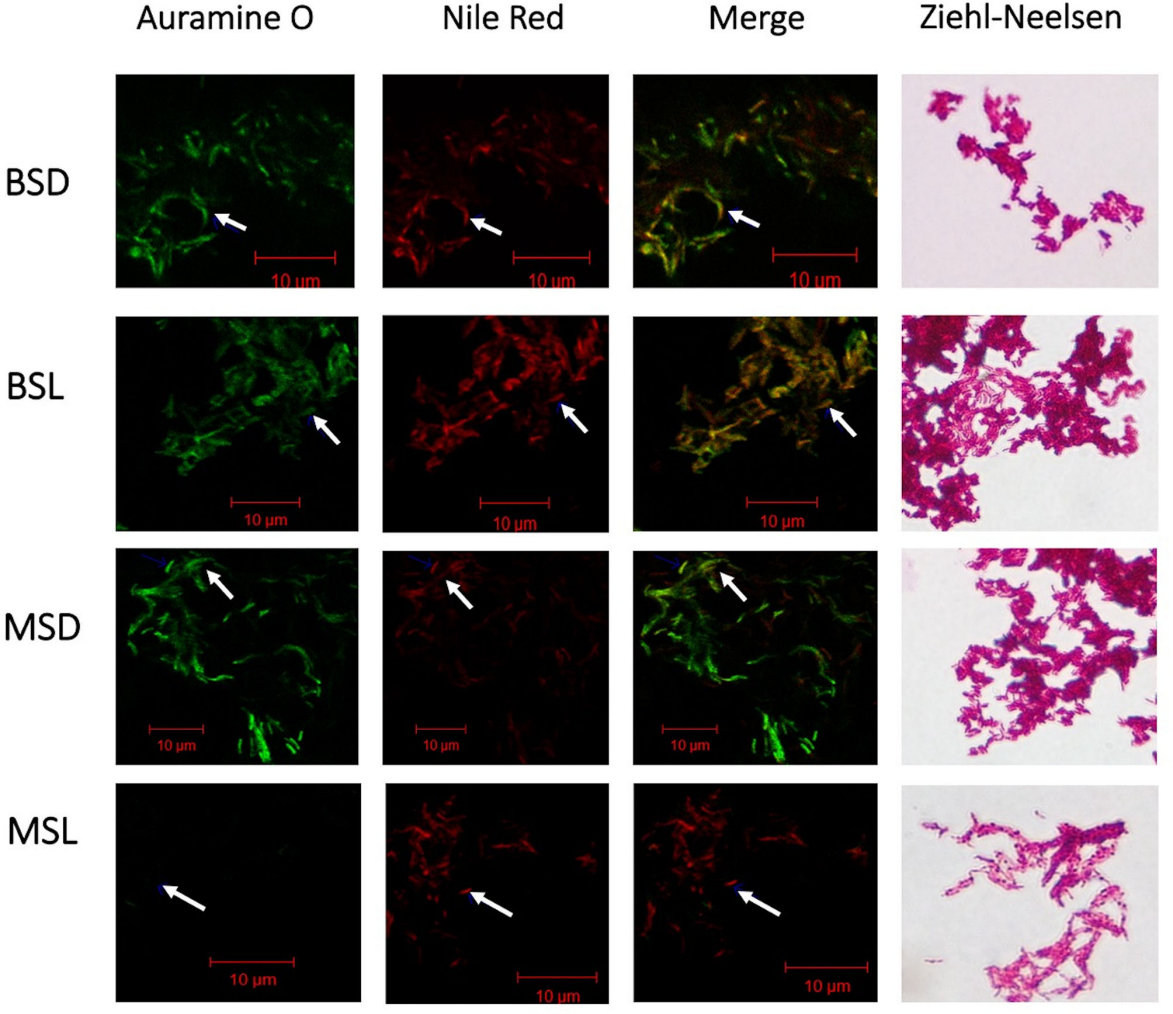
Inactivation of the *BCG_2177c* gene allows neutral-lipid accumulation and decreases acid fastness in *M. bovis* BCG in the presence of fatty acids/cholesterol at the stationary phase. Acid fastness and intracellular lipid accumulation were shown when the mutant strain (mtBCG) was grown at the stationary phase (MSL) in the presence of our lipid mixture. Mycobacteria were stained by auramine O and Nile Red and observed using confocal laser scanning microscopy. BSD (wtBCG cultured in dextrose); BSL (wtBCG cultured in lipids); MSD (mtBCG cultured in dextrose); MSL (mtBCG cultured in lipids). Merge, superposition of green and red fields.

Nile Red stain allowed us to identify changes in the neutral lipid storage in *M. bovis* BCG. These lipids were only evident during the stationary phase of both studied strains (**Figure 5**), independent of the carbon source (dextrose or lipids). Exponential cultures were also stained with auramine and Ziehl–Neelsen, and only few bacilli (~1%) were positively stained by Nile Red (**Figure S4**).8 As expected, the highest quantities of bacilli that accumulate neutral lipids were those cultivated in the presence of cholesterol and fatty acids (BSL and MSL). Also, the mutant strain at the stationary phase (MSL) decreased its ability to be acid fast when it was stained with Auramine O, and no green-fluorescent signal was observed. When these bacilli were colored *via* the Ziehl–Neelsen procedure, they did not present uniform staining (**Figure 5**), showing ghost-appearance bacteria (GAB). There is a possibility that a small quantity of these GAB comprised dead microorganisms, as they are at the stationary phase, but according to the CFU/mL results (see **Figure S1B**), a high percentage of these GAB at the stationary phase are viable cells.

### Monolayer Integrity Kinetics

**Figure 6:**
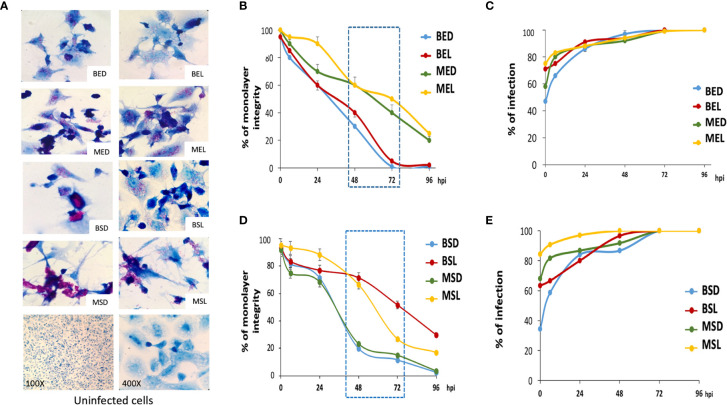
*M. bovis* BCG cultured in lipids produce less damage to macrophages. THP-1 macrophages (3 × 10^5^) were infected with wtBCG or mtBCG (strains at the exponential or stationary phase in the presence of dextrose or lipids) using a MOI of 5:1. After 4 h, cells were washed and incubated with fresh RPMI medium. Infected cells were incubated for 24, 48, 72, and 96 h and stained by Kinyoun stain. **(A)** Panels show representative images of infections at 48 h (400×), and control monolayers without infection. **(B, D)** Cell damage was reported as percentage of monolayer integrity (Helguera-Repetto et al., 2014). Production of cytoplasmic projections and cell vacuolization reported during mycobacterial entry into endothelial cells were also considered as cell damage (Baltierra-Uribe et al., 2014). **(C, E)** Percentages of infection were obtained by counting infected macrophages in 10 fields of the monolayer. wtBCG or mtBCG cultured in dextrose or lipids during the exponential phase (BED, wtBCG dextrose; BEL, wtBCG lipids; MED, mtBCG dextrose; MEL, mtBCG lipids) or the stationary phase (BSD, wtBCG dextrose; BSL, wtBCG lipids; MSD, mtBCG dextrose; MSL, mtBCG lipids).

During the macrophage infection with exponential BCG strains, the cell monolayer integrity was drastically diminished after 48 h. This was observed when macrophages were infected with the wild-type strain grown in both dextrose and lipids (BED and BEL). The integrity of macrophages was maintained longer when they were infected with the mutant strains (MED and MEL) (**Figures 6A, B**).

Additionally, cells coming from the mutant strain cultured at the exponential phase in lipids (MEL) were phagocytosed at the highest rate starting from time 0 (4 h after the first contact of mycobacteria with macrophages), showing a value close to 80% (**Figure 6C**). In contrast, the level of infection produced by the wild-type strain BCG cultivated in dextrose (BED) was the smallest. This specific result was also observed in infections which were caused by the same strain grown in the stationary phase of growth, BSD (**Figure 6E**).

In general, infections produced by mycobacteria grown in lipids from stationary-phase cultures kept the cell integrity longer than those produced by mycobacteria grown in dextrose (**Figures 6A, D**).

### Evaluation of Macrophage–*Mycobacterium bovis* BCG Interaction Kinetics

**Figure 7:**
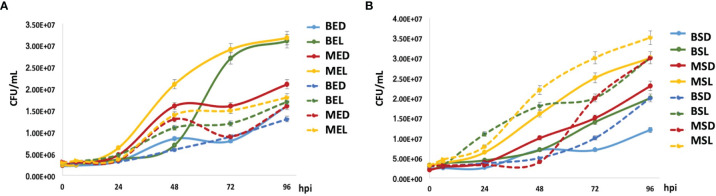
*M. bovis* BCG intracellular growth inside THP-1 macrophages. Mycobacterial multiplication rates were evaluated every 24 h in a 96-h mycobacteria–macrophage interaction kinetic. THP-1 macrophages (5 × 10^5^) were infected with wtBCG or mtBCG strains using a MOI of 5:1. CFUs/mL were calculated (continues lines) and compared with those obtained with each microorganism culture in RPMI medium without macrophages (dotted lines). All experiments were performed in triplicate using wtBCG or mtBCG cultured in dextrose or lipids during the exponential phase **(A)** (BED, wtBCG dextrose; BEL, wtBCG lipids; MED, mtBCG dextrose; MEL, mtBCG lipids), or the stationary phase **(B)** (BSD, wtBCG dextrose; BSL, wtBCG lipids; MSD, mtBCG dextrose; MSL, mtBCG lipids).

*M. bovis* BCG strains (grown at the exponential phase) showed that the mtBCG strain presented the highest level of intracellular replication from 24 hpi onward (in the presence of lipids, MEL) (**Figure 7A**). In general, the mycobacterial multiplication rate was higher than that obtained when bacteria were grown in the absence of macrophages (see discontinuous lines). To be noted, when comparing the same carbon source, the intracellular multiplication of the mutant strain (“MED, MEL” in **Figure 7A**) was higher than that of the wild-type BCG (“BED, BEL” in **Figure 7A**). For example, see the correlation of MED vs. BED or MEL vs. BEL (**Figure 7A**).

In infections produced by mycobacteria grown at stationary phases (**Figure 7B**), the intracellular multiplication of the two strains was apparently controlled by macrophages in all cases. The number of microorganisms in the presence of macrophages was lower than that obtained when the microorganisms were grown in a macrophage-free medium. However, the mutant bacterial culture grown in lipids (MSL) presented the greatest intracellular multiplication rate (**Figure 7B**).

### Cytotoxic Effect in THP-1 Macrophages by *Mycobacterium bovis* BCG

**Figure 8:**
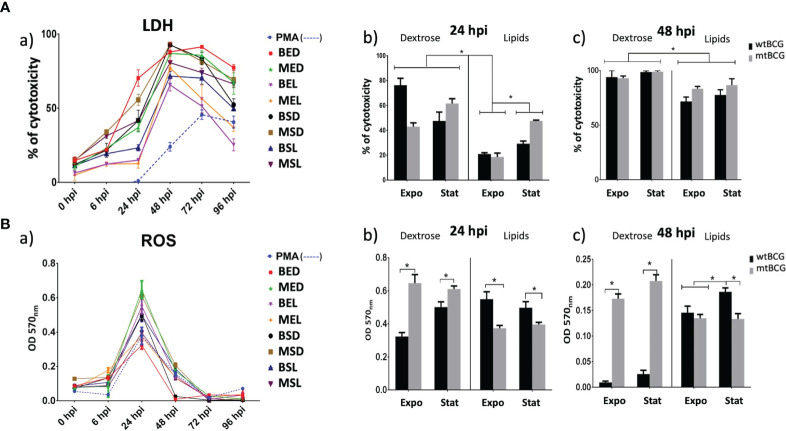
Cytotoxic effect on THP-1 macrophages induced by *M. bovis* BCG strains. THP-1 macrophages (5 × 10^5^) were infected with wtBCG or mtBCG strains using a MOI of 5:1. Cytotoxic effect was measured by **(Aa)** LDH activity released from the cytosol of damaged macrophages and **(Ba)** reactive oxygen species production (ROS) in a 96-h kinetic. Significant changes in LDH activity **(Ab, c)** as well as variations in the ROS production **(Bb, c)** are shown at 24 and 48 hpi. In both LDH and ROS experiments, a positive control of cells in the presence of 100 nM PMA (dotted line) was used. All experiments were performed in triplicate using wtBCG or mtBCG cultured in dextrose or lipids during the exponential phase (BED, wtBCG dextrose; BEL, wtBCG lipids; MED, mtBCG dextrose; MEL, mtBCG lipids) or the stationary phase (BSD, wtBCG dextrose; BSL, wtBCG lipids; MSD, mtBCG dextrose; MSL, mtBCG lipids). *, p < 0.05.

The cytotoxicity effect in THP-1 macrophages was evaluated by measuring the activity of lactate dehydrogenase (LDH) and the production of reactive oxygen species (ROS) during infections. More than 50% of macrophages were damaged from 24 hpi with BED (80%) and MSD (60%) (**Figures 8Aa, b**). In contrast, at the same time, strains cultured in lipids (MEL and BEL) produced very low damage (below 20%) of the monolayer (from 24 hpi) (**Figures 8Aa, b**). At 48 hpi, the cytotoxicity percentage was greater than 50% in all infections. Furthermore, both strains (wtBCG and mtBCG) cultured in the presence of dextrose reached close to 100% toxicity (**Figures 8Aa, c**).

Regarding ROS, the highest production occurred at 24 hpi in all infections (**Figures 8Ba, b**). mtBCG cultured in glucose, either at the exponential or stationary phase (MED, MSD, respectively), was the one that produced the greatest amount of ROS (**Figures 8Ba, b**). Strains that induced the lowest ROS levels at 24 hpi were BED, MEL, and MSL (**Figures 8Ba, b**). It was at this same hpi, 24, and with the exponential-dextrose BCG infections (BED) that the lowest production of ROS was obtained, even lower than that obtained with PMA (**Figure 8Ba**).

Mutant BCG (mtBCG) strains induced more ROS production than wtBCG strains when THP-1 cells were infected with mycobacteria cultured in the presence of dextrose. In the case of strains cultured in the lipid mixture, mtBCG produced fewer ROS levels than wtBCG (**Figure 8Bb**).

Although at 48 hpi the production of ROS decreased sharply (**Figures 8Ba, c**), mainly in wtBCG, under dextrose conditions (BED and BSD), in mtBCG (MED and MSD), it remained high (**Figure 8Bc**). In contrast, in the infections with bacteria cultured in lipids, the production of ROS did not decrease as much as was observed in the wtBCG cultured in dextrose (**Figure 8Bc**).

### Pro-Inflammatory Cytokine (IL-1β, IL-8, and TNF-α) Expression in THP-1 Macrophages Infected With *Mycobacterium bovis BCG*

**Figure 9:**
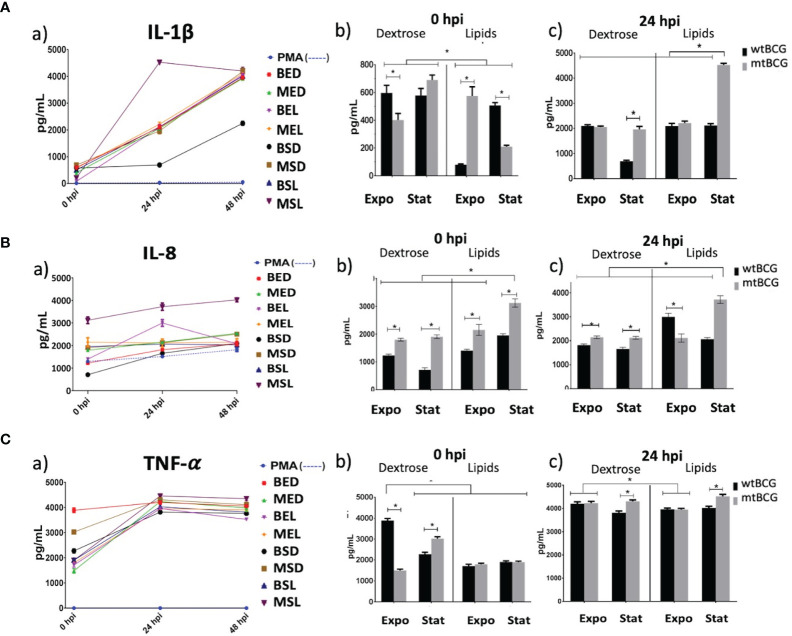
Pro-inflammatory cytokine expression on THP-1 macrophages infected with *M. bovis* BCG strains. Expressions of IL-1β, IL-8, and TNF-α (**A, B, C**, respectively) were determined by ELISA in supernatants collected from different infection times. Significant changes in the concentrations of each cytokine are shown at 0 and 24 hpi (panels **b, c**). In each determination, a positive control (cells overstimulated with 100 nM PMA) was used (dotted lines). All experiments were performed in triplicate using wtBCG or mtBCG cultured in dextrose or lipids during the exponential phase (BED, wtBCG dextrose; BEL, wtBCG lipids; MED, mtBCG dextrose; MEL, mtBCG lipids) or the stationary phase (BSD, wtBCG dextrose; BSL, wtBCG lipids; MSD, mtBCG dextrose; MSL, mtBCG lipids). *, p < 0.05.

IL-1β presented a progressive expression pattern throughout time (**Figure 9Aa**). At time 0, it was observed that wtBCG cultivated in lipids at the exponential phase (BEL) induced a lower production of IL-1β compared with that of the strains cultivated in dextrose (**Figures 9Aa, b**). The highest production of this cytokine occurred at 24 h during infection with the mutant strain grown in lipids (MSL) (**Figures 9Aa, c**). At the end of the kinetic, almost all infections produced the same levels of IL-1β, except for the infection with BSD (**Figures 9Aa, c**).

Both strains, mtBCG and wtBCG (at time 0) cultured in lipids, showed a significant IL-8 expression, being higher in the mutant strain than in wtBCG (**Figures 9Ba, b**). While the MSL strain induced the highest production of this cytokine from 0 to 48 h (**Figures 9Ba, b, c**), the BSD strain induced the lowest levels of it (**Figures 9Ba, b, c**).

Similarly to IL-8 levels, production of TNF-α was presented on a large scale from 0 hpi (levels higher than 1,500 pg/mL) (**Figures 9Ca, b**). There were no significant changes in the starting production of TNF-α (0 hpi) induced by either wild-type or mutant strains cultured in lipids. For this cytokine, BED induced the highest amount of TNF-α at 0 hpi (3,900 pg/mL), while the mutant strain (MED) produced the lowest levels (1,500 pg/mL) (**Figures 9Ca, b**). This low production of TNF-α induced by lipid-cultured strains was increased at 24 hpi, particularly during infection with the mutant strain in the stationary phase (**Figures 9Ca, c**).

### Anti-Inflammatory Cytokine (TGF-β and IL-10) Expression in THP-1 Macrophages Infected With *Mycobacterium bovis BCG*

**Figure 10:**
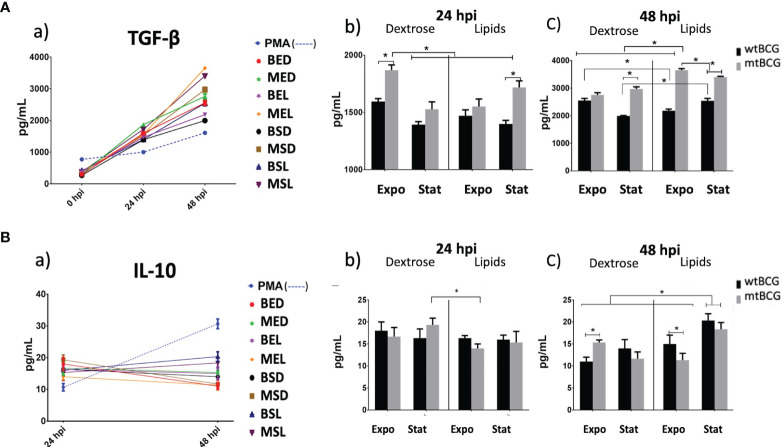
Anti-inflammatory cytokine expression on THP-1 macrophages infected with *M. bovis* BCG strains. Expression of TGF-β and IL-10 (**A, B**, respectively) was determined by ELISA in supernatants collected from different infection times. Significant changes in the concentrations of each cytokine are shown at 24 and 48 hpi (panels **b, c**). IL-10 production at 0 hpi was less than the detection limit of the kit. In each determination, a positive control (cells overstimulated with 100 nM PMA) was used (dotted lines). All experiments were performed in triplicate using wtBCG or mtBCG cultured in dextrose or lipids during the exponential phase (BED, wtBCG dextrose; BEL, wtBCG lipids; MED, mtBCG dextrose; MEL, mtBCG lipids) or the stationary phase (BSD, wtBCG dextrose; BSL, wtBCG lipids; MSD, mtBCG dextrose; MSL, mtBCG lipids). *, p < 0.05.

TGF-β showed a progressive pattern of expression induced by both strains similar to that observed with IL-1β and IL-8 (**Figure 10Aa**). Throughout the whole expression kinetic, levels of TGF-β induced by BCG strains in the presence of lipids were higher than those obtained with bacteria cultured in dextrose (**Figures 10Aa, b, c**). At 24 hpi, MED and MSL strains stimulated a significantly higher production of TGF-β in comparison with wtBCG in the same conditions (**Figure 10Ab**). This was maintained up to 48 hpi, when the highest production of TGF-β was reached during the MEL strain infection (**Figure 10Ac**).

The production of IL-10 was only detected at 24 hpi onward, where values obtained for all infections were similar among all strains but higher than those obtained in the control (PMA 100 nM). The only significant difference was observed between the infection with the mutant strain cultured in dextrose (MSD) and the same strain cultured in lipids in the exponential phase (MEL), the latter presenting the lowest levels of IL-10 (**Figures 10Aa, b**). At 48 hpi, strains cultured in lipids at the stationary phase (BSL and MSL) induced the highest expression of IL-10 (**Figure 10A**). Comparing only exponential cultures in lipids, the mtBCG strain showed a higher expression of IL-10 than that produced with wtBCG (**Figure 10Ac**).

## Discussion

Our group has previously found a relationship between the inhibition (by transposition of Tngfp in the *BCG*_*2177c* gene) of a TetR-like repressor and the overexpression of some genes that participate in the metabolism of cholesterol (Otal et al., 2017). Therefore, in this study our main aim was to elucidate some environmental and host factors that might control the function of this putative repressor gene. Knowledge of this could contribute to further understanding the role of lipids in the mycobacteria host–pathogen relationship, particularly in the physiology of this BCG vaccine strain.

### The Fatty Acid/Cholesterol Mixture Enhances Gene Expression in *M. bovis* BCG (“Lipid Signature”)

In comparison to dextrose, we found an overexpression (number of reads) of genes in both wild-type and mutant BCGs (**Figure 2**, columns 1–4) when these mycobacteria were cultured in lipids. As these results were similar to those observed in *Mtb* (Del Portillo et al., 2019), we can assume that the overexpression in the presence of lipids supports the important role of these molecules for pathogenic mycobacteria physiology and its global metabolism.

Among the vast numbers of differentially expressed genes in the environmental conditions studied in this work, we have further examined 20 genes that were significantly overexpressed in the presence of lipids (regardless of the type of the strain or their growth phase). We called this group of genes the “lipid signature” of *M. bovis* BCG. This signature included genes related to mycobacterial transcriptional regulation, intracellular-redox balance maintenance, and some hypothetical proteins (**Table 1**).

Four transcriptional regulator genes, ***BCG*_*RS20155*, *BCG_RS16075*, *BCG_RS07550*, and *BCG_RS10355*** were detected. Among these, *BCG*_*RS20155* displayed a significantly high expression in most conditions (**Figure 3**). This is a gene homologous to *Rv3862c*, which in *Mtb* codes for *whi*B6 (Solans et al., 2014). In view of the fact that WhiB proteins have two functions, acting either as global transcriptional regulators or as controller of the bacterial redox state (Alam et al., 2009), in our model *BCG*_*RS20155* overexpression might be related to the regulation of the intracellular redox balance. The overexpression of the other 16 genes (according to their function) presented in **Table 1** (first column) could indicate that lipids might prepare mycobacteria to persist under the stress environment found inside their host, as previously reported for *Mtb*, BCG, and other microorganisms (Dubnau et al., 2000; Turner et al., 2001; Starck et al., 2004; Gazdik et al., 2009; Sawers et al., 2016).

None of our lipid signature genes are members of the KstR or KstR2 regulon. However, we found a similar overexpression of genes *BCG_RS07550* (*Rv1395*), *BCG_RS20155* (*Rv3862c*), *BCG_RS02160* (*Rv0384c*), *BCG_RS02005* (*Rv0352*), and *BCG_RS02000* (*Rv0351*), with their *Mtb* orthologs reported by Schnappinger et al. (2003), where a palmitic acid signature gene was described. Hence, we can assume that the other 15 genes of our lipid signature were overexpressed as the result of the combination of stearic and oleic acids with cholesterol. Assimilation of host-derived lipids (fatty acids and cholesterol) is essential for *Mtb* persistence. This mechanism in this bacterium depends on Mce1 and Mce4 transporters through LucA (*Rv3723*) regulation (Nazarova et al., 2017).

We are aware that having a complemented strain of our mutant BCG would be ideal in order to confirm our results, and undoubtedly, this should be done in the future. Nevertheless, the results found by Nazarova et al. (2017) about the global expression pattern of a *luc*A mutant of *Mtb* infecting macrophages are encouraging for us. They found that such a mutant strain downregulated the *tet*R (*BCG_2177c*) repressor gene and, in turn, also downregulated some genes related to mycobacterial lipid metabolism, including *clp*B, *dna*J, and *grp*E, which correlates with our data. When *tet*R is lacking, the expression of these three genes appears to be increased, and the same result was found and reported in our work.

The presence of 64 ncRNA in the genome of *M. bovis* BCG has previously been reported, and their differential expression has only been shown in cultures with glycerol-oleic acid and dextrose as main carbon sources (Haning et al., 2014). The ncRNA, *ncBCG1323Ac* gene, was overexpressed in the presence of lipids particularly in the wild-type strain (BSL) and when the mutant strain was cultured in dextrose at the stationary phase (MSD) (**Figure 4**). Similarly, this *M. bovis* BCG ncRNA has been reported to be overexpressed at the stationary phase of growth, at acidic pH, and under hypoxic conditions in the presence of glycerol–glucose–oleic acid as carbon sources (DiChiara et al., 2010). Therefore, we can propose that this particular ncRNA should be further investigated as an important regulator for the survival of this microorganism under different stress conditions (hypoxia, starvation, acidic pH, etc.).

### Neutral Lipid Storage in *M. bovis BCG*

We found that wild-type and mutant strains of BCG stored a large amount of neutral lipids when they were cultured in the presence of a lipid mixture (fatty acids/cholesterol). This phenomenon has also been described in *Mtb*, when it contacts THP-1 macrophages, and has been associated with the use, by this microorganism, of lipids present in foamy macrophages (triacylglycerides) (Daniel et al., 2011; Fozo and Rucks, 2016; Santucci et al., 2016). Likewise, a loss of acid-fast resistance has also been reported in *Mtb* bacilli as they store neutral lipids and begin to enter a non-replicative state (Daniel et al., 2011).

Since the mutant BCG strain is not stained with auramine O in the presence of lipids (MSL) and is only partially stained with the Ziehl–Neelsen technique at the stationary phase of growth, we suggest that mutation of the *BCG_2177c* gene interferes during the auramine staining, as has been reported during macrophage infection with *Mtb* (Daniel et al., 2011).

Additionally, the presence of fatty acids in the medium probably avoids the formation of cord-like structures in the mutant strain, reported previously when mtBCG was cultured in a medium with cholesterol as a sole carbon source (Otal et al., 2017). In our work, the presence of fatty acid may contribute to reducing the cholesterol effects in the cell envelope of BCG, and therefore, in its staining properties. This is also confirmed by our BCG transcriptome results (data not shown), where *fbp*A and *fbp*B gene expressions (related to the synthesis of trehalose dimycolate, the cord factor in *Mtb*) were diminished in the mutant strain when in the presence of the fatty acid/cholesterol mix.

### mtBCG Cultured in Lipids Induced Less Cytotoxic Damage of THP-1 Macrophages

It is known that the extracellular levels of LDH increase when cell integrity is disrupted under oxidative stress conditions (Corleis et al., 2012; Kumar et al., 2018). Here, for both strains and in all conditions, we found that oxidative stress occurred before 24 hpi, and therefore LDH levels were higher after this time (**Figure 8**). The lowest ROS levels were found during infections with the mtBCG strain cultured in lipids (**Figure 8B**). Surprisingly, this strain cultured in dextrose exhibited the highest levels of ROS in all experiments. All previous results support the critical role of lipids in conferring advantages in the process of internalization and survival of mycobacteria during chronic infection, a process that has been studied in *Mtb*, where protective and detoxification mechanisms to maintain cytoplasmic redox balance are fundamental for intracellular survival (Mendum et al., 2015). During this adaptation process, *Mtb* modulates lipid biosynthesis, lipid storage, and expression of virulence factors as a dissipative mechanism for mycobacteria survival (Mavi et al., 2020). Strains cultured in lipids reached a high intracellular growth rate (based on the percentage of infection and CFU/mL, **Figures 6B, C**, **7**) when they were in contact with macrophages. Considering the percentage found in the infection data, we can propose that the mutation in the *BCG_2177c* gene, together with the presence of lipids, provokes not only the phagocytosis of mycobacteria but also their survival inside macrophages for a longer time than strains grown in dextrose.

When macrophages were infected by the mutant strain, we found a higher amount of CFUs and better cell integrity than with the wild-type strain. These results correlate with low levels of cytotoxicity and ROS production found in our mutant. Taken together, our results would indicate that our mutant strain, while in the presence of high ROS levels, may modulate the intracellular redox balance and survive longer inside macrophages. This agrees with a recent report carried out by de Lima et al. (2021) where it was found that *M. smegmatis* grown in cholesterol persists intracellularly for a longer time causing less damage than when it grows in dextrose or glycerol. Our data also reveal that, after growing in a lipid mixture, the infection of THP-1 macrophages by *M. bovis BCG* strains (wild-type and mutant) somehow results in a modulation of the immune response to facilitate their survival inside the host. An in-depth investigation of the processes that lead to revealing this modulation by fatty acids and cholesterol, in particular, would allow further insights in understanding the host–pathogen relationship in mycobacteria.

### Pro-Inflammatory and Anti-Inflammatory Response of THP-1 Macrophages, During Infections With BCG in a Lipid Scenario

TNF-α, IL-1β, and IL-8 are pro-inflammatory cytokines of the innate immune response which are involved in the formation of the *Mtb* granuloma (Lyon and Rossman, 2017; Mishra et al., 2017). In our work, we found overexpression of TNF-α, IL-1β, and IL-8 in most infections, which agrees with the results of Tripathi et al. (2020).These authors found that ClpB induced an inflammatory response in THP-1 macrophages during *Mtb* infection, increasing the levels of TNF-α and IL-6 after 24 h postinfection (Tripathi et al., 2020). In this respect, we found that the overexpression of the ClpB system (through the overexpression of *clpB*, *dnaJ*, and *grpE* genes, see **Table 1**) with the mutant strain cultured in lipids at the stationary phase (MSL) induced the highest levels of those three pro-inflammatory cytokines at 24 hpi (**Figure 9**).

Even if the previously reported capacity of *M. bovis* BCG to induce strong levels of TNF-α during THP-1 infection (Riendeau and Kornfeld, 2003) was preserved in both mutant and wild-type strains, the presence of lipids delayed its induction until 24 hpi (**Figure 9C**). Thus, we hypothesize that the inhibition of the *BCG_2177c* gene and the presence of lipids in the media induced a strong response focused on maintaining the intracellular redox balance, which also promoted an establishment of and control of a pro-inflammatory response during the early macrophage infection with this strain.

In the same way, MSL (mutant strain/stationary phase/lipids) induced one of the lowest ROS productions and very little damage to macrophages was observed (cell integrity was conserved at 73% up to 48 hpi). It has been reported that anti-inflammatory cytokines such as IL-10 and TGF-β decrease the production of pro-inflammatory cytokines and the oxidative response (Lee et al., 2009). The main function of these molecules is to control tissue damage during the immune response against *Mtb* (Mayer-Barber and Sher, 2015). In our work, the mutant strain induced an anti-inflammatory response through TGF-β. In all studied conditions, TGF-β levels were higher in the mutant strain than in the wild-type (**Figure 10A**). With the mutant strain, the lowest level of this cytokine was produced by this strain in the exponential phase of growth (MEL). Therefore, the hypothesis might be that with a MEL infection, one of the roles of TGF-β would be to avoid tissue damage generated by TNF-α. This latter cytokine, in the presence of a pro-inflammatory environment as well as an anti-inflammatory environment, could produce necrosis and in turn cause a higher cell multiplication rate of the mutant BCG, similar to that observed in *Mtb* and in some non-tuberculous mycobacteria (Hernandez-Pando et al., 2009; Helguera-Repetto et al., 2014).

At the same time, the low ROS production at 24 hpi, mainly in the mutant strain (MEL and MSL), may be provoked by the overproduction of an anti-inflammatory response mediated by the overexpression of TGF-β. This effect was contrary to that observed in cultures derived from dextrose. In previous studies, it has been found that peripheral blood monocytes from patients with active TB produce high levels of TGF-β (Toossi et al., 1995; Wu et al., 2012). A similar effect was found in THP-1 infections with *M. celatum*, where this species was able to evade the production of the respiratory burst and manage to survive intracellularly through TGF-β production (Helguera-Repetto et al., 2014). In this way, the mutant strain controls the cell damage that TNF-α might cause but generates a major mycobacterium growing rate.

**Figure 11:**
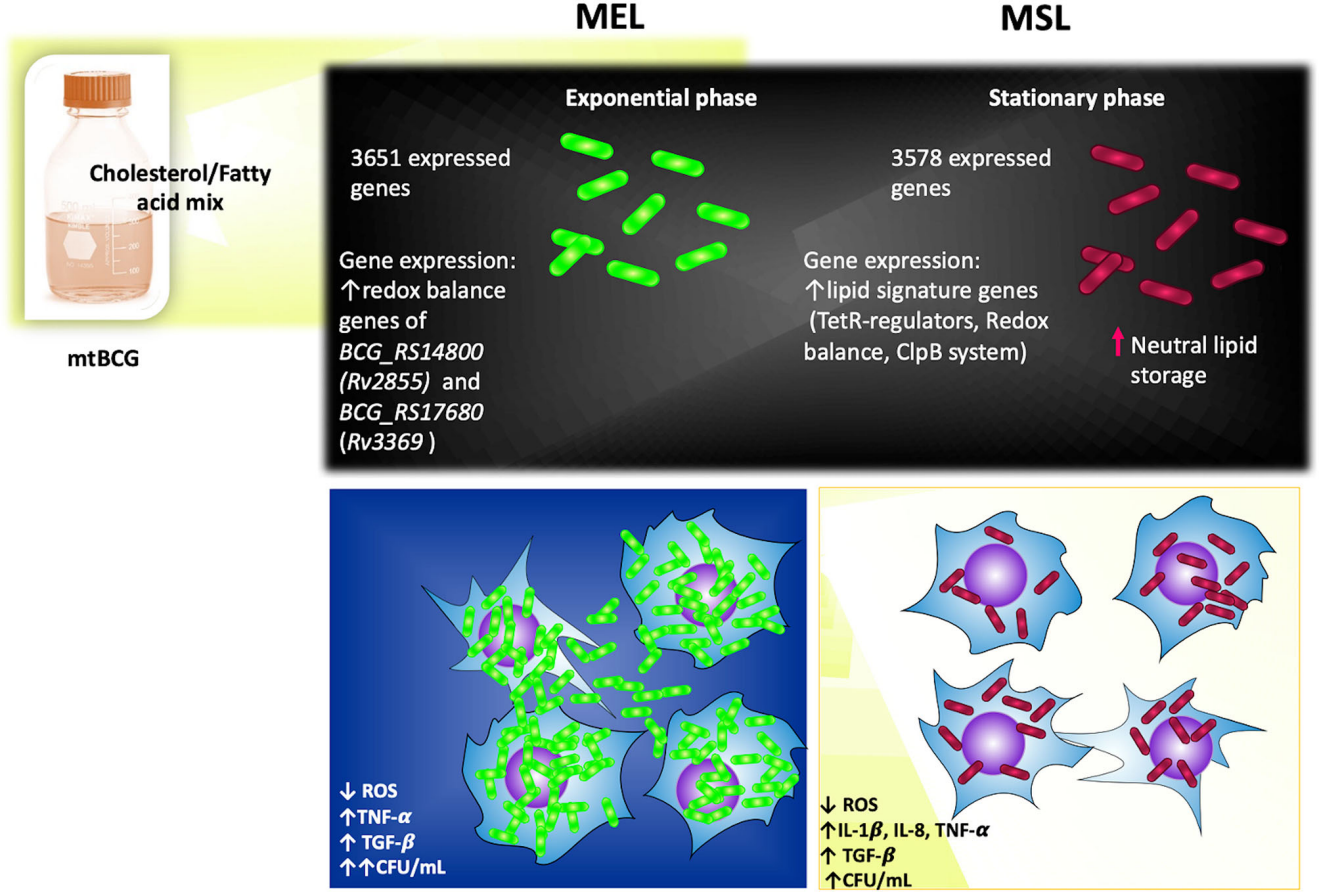
Effects of lipids and *BCG*_*2177c* gene inactivation on the physiology of *M. bovis* BCG (Summary). See the text in Discussion (in the summary paragraph) for details.

In summary (**Figure 11**), the mutation of the *BCG_2177c* gene, together with the presence of lipids, favors the intracellular storage of neutral lipids in the stationary phase of *M. bovis* BCG (MSL). This particular environment also leads this mycobacterium (both MEL and MSL) to overexpress some genes related to maintaining an intracellular redox balance. Although a lower number of genes were expressed in the MSL strain (in comparison to MEL), more representative genes for the “lipid signature of *M. bovis*” were found in it, such as *tet*-R transcriptional regulators genes, redox balance genes, and ClpB system genes. During the initial steps of infections with MEL and MSL, mycobacteria were able to evade the oxidative mechanisms of the innate immune response (ROS production by macrophages) and stimulate a pro-inflammatory environment (TNF-α, IL-1β, and IL-8). Subsequently, those strains induced a high production of TGF-β, avoiding low damage to host cells and, in turn stimulated their intracellular persistence.

## Concluding Remarks

Lipids play a crucial role during infections with species of the *Mycobacterium tuberculosis* complex (MTBC) and have been proposed as key regulators of their global metabolism, inducing genotypic and phenotypic changes that are not limited to structural function. Lipid metabolism has been studied in *Mtb* models using fatty acids or cholesterol at normoxia and hypoxia conditions, where some key regulators have been proposed. Some of them (Mce3R, FdmR, KstR, KstR2, and Fad35R) are members of the TetR-like transcriptional regulators.

When fatty acids/cholesterol were present in the culture medium, we found a more active transcriptome which is more related to counteracting intracellular reductive stress. Additionally, within a lipidic scenario, the inactivation of *BCG*_*2177c* (*Rv2160A*) did not interfere with cell growth but probably affected the composition of the cell envelope. These actions have consequences during infection of THP-1 macrophages, making the mutant strain better at persisting intracellularly, modulating the immune response to over-synthesize anti-inflammatory TGF-β and diminishing cytotoxic damages.

## Data Availability Statement

The datasets presented in this study can be found in online repositories. The names of the repository/repositories and accession number(s) can be found below: https://www.ncbi.nlm.nih.gov/geo/, GSE175579.

## Author Contributions

SR-G and JG-y-M conceived and designed the study. LG-M and JA carried out the experiments. MA, AH-R, PP, MG, JC-C, IO, and CM analyzed the data. JMA and AM-T helped to analyze the RNA-seq data. LG-M and MA performed the statical analysis. LG-M developed **Figure 11** (Effects of lipids and *BCG*_*2177c* gene inactivation on the physiology of *M. bovis* BCG). LG-M, MA, and JG-y-M wrote the first draft of the manuscript. All authors contributed to manuscript revision and read and approved the submitted version.

## Funding

This work was supported by grant CB-2015-01-255181 (SR-G) from Consejo Nacional de Ciencia y Tecnología, México, and grants SIP20221037 (SR-G) and SIP20220091 (JC-C), from the National Polytechnic Institute (IPN), Mexico. It was also supported by Fondo Europeo de Desarrollo Regional (FEDER), una manera de hacer Europa, Proyecto código PI19/00666 (MG), Spain.

## Conflict of Interest

The authors declare that the research was conducted in the absence of any commercial or financial relationships that could be construed as a potential conflict of interest.

## Publisher’s Note

All claims expressed in this article are solely those of the authors and do not necessarily represent those of their affiliated organizations, or those of the publisher, the editors and the reviewers. Any product that may be evaluated in this article, or claim that may be made by its manufacturer, is not guaranteed or endorsed by the publisher.

## Abbreviations

wtBCG, wild-type *Mycobacterium bovis* BCG Pasteur 1173P2; mtBCG, a mutant strain of *M. bovis* BCG obtained by transposition of the *Tngfp* in the *BCG_2177c* gene, that codes for a putative TetR repressor; BED, wtBCG cultured at the exponential phase in dextrose; BEL, wtBCG cultured at the exponential phase in lipids; MED, mtBCG cultured at the exponential phase in dextrose; MEL, mtBCG cultured at the exponential phase in lipids; BSD, wtBCG cultured at the stationary phase in dextrose; BSL, wtBCG cultured at the stationary phase in lipids; MSD, mtBCG cultured at the stationary phase in dextrose; MSL, mtBCG cultured at the stationary phase in lipids.

## References

[B1] Aguilar-Ayala D. A. Tilleman L. Van Nieuwerburgh F. Deforce D. Palomino J. C. Vandamme P.. (2017). The Transcriptome of *Mycobacterium tuberculosis* in a Lipid-Rich Dormancy Model Through RNAseq Analysis. Sci. Rep. 7, 17665. doi: 10.1038/s41598-017-17751-x 29247215 PMC5732278

[B2] Alam M. S. Garg S. K. Agrawal P. (2009). Studies on Structural and Functional Divergence Among Seven WhiB Proteins of *Mycobacterium tuberculosis* H37Rv. FEBS J. 276, 76–93. doi: 10.1111/j.1742-4658.2008.06755.x 19016840

[B3] Anand S. Singh V. Singh A. K. Mittal M. Datt M. Subramani B.. (2012). Equilibrium Binding and Kinetic Characterization of Putative Tetracycline Repressor Family Transcription Regulator Fad35R From *Mycobacterium tuberculosis* . FEBS J. 279, 3214–3228. doi: 10.1111/j.1742-4658.2012.08707.x 22805491

[B4] Ayyappan J. P. Ganapathi U. Lizardo K. Vinnard C. Subbian S. Perlin D. S.. (2019). Adipose Tissue Regulates Pulmonary Pathology During TB Infection. MBio 10 (2), e02771–18. doi: 10.1128/mBio.02771-18 PMC6469978 30992360

[B5] Balhana R. J. C. Singla A. Sikder M. H. Withers M. Kendall S. L. (2015). Global Analyses of TetR Family Transcriptional Regulators in Mycobacteria Indicates Conservation Across Species and Diversity in Regulated Functions. BMC Genomics 16, 479. doi: 10.1186/s12864-015-1696-9 26115658 PMC4482099

[B6] Baltierra-Uribe S. L. García-Vásquez M. de J. Castrejón-Jiménez N. S. Estrella-Piñón M. P. Luna-Herrera J. García-Pérez B. E. (2014). Mycobacteria Entry and Trafficking Into Endothelial Cells. Can. J. Microbiol. 60, 569–577. doi: 10.1139/cjm-2014-0087 25113069

[B7] Bertram R. Hillen W. (2008). The Application of Tet Repressor in Prokaryotic Gene Regulation and Expression. Microb. Biotechnol. 1, 2–16. doi: 10.1111/j.1751-7915.2007.00001.x 21261817 PMC3864427

[B8] Chang J. C. Miner M. D. Pandey A. K. Gill W. P. Harik N. S. Sassetti C. M.. (2009). Igr Genes and *Mycobacterium tuberculosis* Cholesterol Metabolism. J. Bacteriol. 191, 5232–5239. doi: 10.1128/JB.00452-09 19542286 PMC2725594

[B9] Corleis B. Korbel D. Wilson R. Bylund J. Chee R. Schaible U. E. (2012). Escape of *Mycobacterium tuberculosis* From Oxidative Killing by Neutrophils. Cell. Microbiol. 14, 1109–1121. doi: 10.1111/j.1462-5822.2012.01783.x 22405091

[B10] Daniel J. Maamar H. Deb C. Sirakova T. D. Kolattukudy P. E. (2011). *Mycobacterium tuberculosis* Uses Host Triacylglycerol to Accumulate Lipid Droplets and Acquires a Dormancy-Like Phenotype in Lipid-Loaded Macrophages. PloS Pathog. 7, e1002093. doi: 10.1371/journal.ppat.1002093 21731490 PMC3121879

[B11] de Lima J. B. da Silva Fonseca L. P. Xavier L. P. de Matos Macchi B. Cassoli J. S. da Silva E. O.. (2021). Culture of *Mycobacterium smegmatis* in Different Carbon Sources to Induce *In Vitro* Cholesterol Consumption Leads to Alterations in the Host Cells After Infection: A Macrophage Proteomics Analysis. Pathogens 10, 662. doi: 10.3390/pathogens10060662 34071265 PMC8230116

[B12] Del Portillo P. García-Morales L. Menéndez M. C. Anzola J. M. Rodríguez J. G. Helguera-Repetto A. C.. (2019). Hypoxia Is Not a Main Stress When *Mycobacterium tuberculosis* Is in a Dormancy-Like Long-Chain Fatty Acid Environment. Front. Cell. Infect. Microbiol. 8. doi: 10.3389/fcimb.2018.00449 PMC6333855 30687646

[B13] DiChiara J. M. Contreras-Martinez L. M. Livny J. Smith D. McDonough K. A. Belfort M. (2010). Multiple Small RNAs Identified in *Mycobacterium bovis* BCG are Also Expressed in *Mycobacterium tuberculosis* and *Mycobacterium smegmatis* . Nucleic Acids Res. 38, 4067–4078. doi: 10.1093/nar/gkq101 20181675 PMC2896511

[B14] Dong W. Nie X. Zhu H. Liu Q. Shi K. You L.. (2021). Mycobacterial Fatty Acid Catabolism is Repressed by FdmR to Sustain Lipogenesis and Virulence. Proc. Natl. Acad. Sci. 118 (16), e2019305118. doi: 10.1073/pnas.2019305118 33853942 PMC8072231

[B15] Dubnau E. Chan J. Raynaud C. Mohan V. P. Lanéelle M. Yu K.. (2000). Oxygenated Mycolic Acids are Necessary for Virulence of *Mycobacterium tuberculosis* in Mice. Mol. Microbiol. 36, 630–637. doi: 10.1046/j.1365-2958.2000.01882.x 10844652

[B16] Edgar R. Domrachev M. Lash A. E. (2002). Gene Expression Omnibus: NCBI Gene Expression and Hybridization Array Data Repository. Nucleic Acids Res. 30, 207–210. doi: 10.1093/nar/30.1.207 11752295 PMC99122

[B17] Fine-Coulson K. Reaves B. J. Karls R. K. Quinn F. D. (2012). The Role of Lipid Raft Aggregation in the Infection of Type II Pneumocytes by *Mycobacterium tuberculosis* . PloS One 7, e45028. doi: 10.1371/journal.pone.0045028 23024786 PMC3443240

[B18] Fozo E. M. Rucks E. A. (2016). The Making and Taking of Lipids: The Role of Bacterial Lipid Synthesis and the Harnessing of Host Lipids in Bacterial Pathogenesis. Adv. Microb. Physiol. 69, 51–155. doi: 10.1016/bs.ampbs.2016.07.001 27720012

[B19] García-Fernández E. Medrano F. J. Galán B. García J. L. (2014). Deciphering the Transcriptional Regulation of Cholesterol Catabolic Pathway in Mycobacteria: Identification of the Inducer of KstR Repressor. J. Biol. Chem. 289, 17576–17588. doi: 10.1074/jbc.M113.545715 24802756 PMC4067193

[B20] Gazdik M. A. Bai G. Wu Y. McDonough K. A. (2009). Rv1675c (Cmr) Regulates Intramacrophage and Cyclic AMP-Induced Gene Expression in *Mycobacterium tuberculosis*-Complex Mycobacteria. Mol. Microbiol. 71, 434–448. doi: 10.1111/j.1365-2958.2008.06541.x 19040643 PMC2845544

[B21] Haning K. Cho S. H. Contreras L. M. (2014). Small RNAs in Mycobacteria: An Unfolding Story. Front. Cell. Infect. Microbiol. 4. doi: 10.3389/fcimb.2014.00096 PMC4109619 25105095

[B22] Helguera-Repetto A. C. Chacon-Salinas R. Cerna-Cortes J. F. Rivera-Gutierrez S. Ortiz-Navarrete V. Estrada-Garcia I.. (2014). Differential Macrophage Response to Slow- and Fast-Growing Pathogenic Mycobacteria. BioMed. Res. Int. 2014, 916521. doi: 10.1155/2014/916521 24949482 PMC4052160

[B23] Hernandez-Pando R. Orozco H. Aguilar D. (2009). Factors That Deregulate the Protective Immune Response in Tuberculosis. Arch. Immunol. Ther. Exp. (Warsz) 57, 355–367. doi: 10.1007/s00005-009-0042-9 19707720

[B24] Houben R. M. G. J. Dodd P. J. (2016). The Global Burden of Latent Tuberculosis Infection: A Re-Estimation Using Mathematical Modelling. PloS Med. 13, e1002152. doi: 10.1371/journal.pmed.1002152 27780211 PMC5079585

[B25] Kendall S. L. Burgess P. Balhana R. Withers M. Bokum A. Lott J. S.. (2010). Cholesterol Utilization in Mycobacteria Is Controlled by Two TetR-Type Transcriptional Regulators: kstR and Kstr2. Microbiology 156, 1362–1371. doi: 10.1099/mic.0.034538-0 20167624 PMC3068626

[B26] Kendall S. L. Withers M. Soffair C. N. Moreland N. J. Gurcha S. Sidders B.. (2007). A Highly Conserved Transcriptional Repressor Controls a Large Regulon Involved in Lipid Degradation in *Mycobacterium smegmatis* and *Mycobacterium tuberculosis* . Mol. Microbiol. 65, 684–699. doi: 10.1111/j.1365-2958.2007.05827.x 17635188 PMC1995591

[B27] Kim M. J. Wainwright H. C. Locketz M. Bekker L. G. Walther G. B. Dittrich C.. (2010). Caseation of Human Tuberculosis Granulomas Correlates With Elevated Host Lipid Metabolism. EMBO Mol. Med. 2, 258–274. doi: 10.1002/emmm.201000079 20597103 PMC2913288

[B28] Kopylova E. Noé L. Touzet H. (2012). SortMeRNA: Fast and Accurate Filtering of Ribosomal RNAs in Metatranscriptomic Data. Bioinformatics 28, 3211–3217. doi: 10.1093/bioinformatics/bts611 23071270

[B29] Kumar A. Bose M. Brahmachari V. (2003). Analysis of Expression Profile of Mammalian Cell Entry (Mce) Operons of *Mycobacterium tuberculosis* . Infect. Immun. 71, 6083–6087. doi: 10.1128/IAI.71.10.6083-6087.2003 14500535 PMC201107

[B30] Kumar P. Nagarajan A. Uchil P. D. (2018). Analysis of Cell Viability by the Lactate Dehydrogenase Assay. Cold Spring Harb. Protoc. 6, 465–68. doi: 10.1101/pdb.prot095497 29858337

[B31] Lara J. Diacovich L. Trajtenberg F. Larrieux N. Malchiodi E. L. Fernández M. M.. (2020). *Mycobacterium tuberculosis* FasR Senses Long Fatty Acyl-CoA Through a Tunnel and a Hydrophobic Transmission Spine. Nat. Commun. 11, 3703. doi: 10.1038/s41467-020-17504-x 32710080 PMC7382501

[B32] Lee J. Hartman M. Kornfeld H. (2009). Macrophage Apoptosis in Tuberculosis. Yonsei Med. J. 50, 1–11. doi: 10.3349/ymj.2009.50.1.1 19259342 PMC2649858

[B33] Lovewell R. R. Sassetti C. M. VanderVen B. C. (2016). Chewing the Fat: Lipid Metabolism and Homeostasis During *M. tuberculosis* Infection. Curr. Opin. Microbiol. 29, 30–36. doi: 10.1016/j.mib.2015.10.002 26544033

[B34] Lyon S. M. Rossman M. D. (2017). Pulmonary Tuberculosis. Microbiol. Spectr. 5, 345–394. doi: 10.1128/microbiolspec.TNMI7-0032-2016 PMC11687454 28185620

[B35] Mavi P. S. Singh S. Kumar A. (2020). Reductive Stress: New Insights in Physiology and Drug Tolerance of *Mycobacterium* . Antioxid. Redox Signal. 32, 1348–1366. doi: 10.1089/ars.2019.7867 31621379

[B36] Mayer-Barber K. D. Sher A. (2015). Cytokine and Lipid Mediator Networks in Tuberculosis. Immunol. Rev. 264, 264–275. doi: 10.1111/imr.12249 25703565 PMC4339232

[B37] Mendum T. A. Wu H. Kierzek A. M. Stewart G. R. (2015). Lipid Metabolism and Type VII Secretion Systems Dominate the Genome Scale Virulence Profile of *Mycobacterium tuberculosis* in Human Dendritic Cells. BMC Genomics 16, 372. doi: 10.1186/s12864-015-1569-2 25956932 PMC4425887

[B38] Menzies D. Adjobimey M. Ruslami R. Trajman A. Sow O. Kim H.. (2018). Four Months of Rifampin or Nine Months of Isoniazid for Latent Tuberculosis in Adults. N. Engl. J. Med. 379, 440–453. doi: 10.1056/NEJMoa1714283 30067931

[B39] Mishra B. B. Lovewell R. R. Olive A. J. Zhang G. Wang W. Eugenin E.. (2017). Nitric Oxide Prevents a Pathogen-Permissive Granulocytic Inflammation During Tuberculosis. Nat. Microbiol. 2, 17072. doi: 10.1038/nmicrobiol.2017.72 28504669 PMC5461879

[B40] Nazarova E. V. Montague C. R. La T. Wilburn K. M. Sukumar N. Lee W.. (2017). Rv3723/LucA Coordinates Fatty Acid and Cholesterol Uptake in *Mycobacterium tuberculosis* . Elife 6, 1–22. doi: 10.7554/eLife.26969 PMC5487216 28708968

[B41] Orth P. Schnappinger D. Hillen W. Saenger W. Hinrichs W. (2000). Structural Basis of Gene Regulation by the Tetracycline Inducible Tet Repressor-Operator System. Nat. Struct. Biol. 7, 215–219. doi: 10.1038/73324 10700280

[B42] Otal I. Pérez-Herrán E. Garcia-Morales L. Menéndez M. C. Gonzalez-y-Merchand J. A. Martín C.. (2017). Detection of a Putative TetR-Like Gene Related to *Mycobacterium bovis* BCG Growth in Cholesterol Using a Gfp-Transposon Mutagenesis System. Front. Microbiol. 8. doi: 10.3389/fmicb.2017.00315 PMC5337628 28321208

[B43] Ouellet H. Johnston J. B. de Montellano P. R. (2011). Cholesterol Catabolism as a Therapeutic Target in *Mycobacterium tuberculosis* . Trends Microbiol. 19, 530–539. doi: 10.1016/j.tim.2011.07.009 21924910 PMC3205253

[B44] Pandey A. K. Sassetti C. M. (2008). Mycobacterial Persistence Requires the Utilization of Host Cholesterol. Proc. Natl. Acad. Sci. U. S. A. 105, 4376–4380. doi: 10.1073/pnas.0711159105 18334639 PMC2393810

[B45] Prosser G. Brandenburg J. Reiling N. Barry C. E. Wilkinson R. J. Wilkinson K. A. (2017). The Bacillary and Macrophage Response to Hypoxia in Tuberculosis and the Consequences for T Cell Antigen Recognition. Microbes Infect. 19, 177–192. doi: 10.1016/j.micinf.2016.10.001 27780773 PMC5335906

[B46] Ramos J. L. Martinez-Bueno M. Molina-Henares A. J. Teran W. Watanabe K. Zhang X.. (2005). The TetR Family of Transcriptional Repressors. Microbiol. Mol. Biol. Rev. 69, 326–356. doi: 10.1128/MMBR.69.2.326-356.2005 15944459 PMC1197418

[B47] Riendeau C. J. Kornfeld H. (2003). THP-1 Cell Apoptosis in Response to Mycobacterial Infection. Infect. Immun. 71, 254–259. doi: 10.1128/IAI.71.1.254-259.2003 12496173 PMC143334

[B48] Rodríguez J. G. Hernández A. C. Helguera-Repetto C. Aguilar Ayala D. Guadarrama-Medina R. Anzóla J. M.. (2014). Global Adaptation to a Lipid Environment Triggers the Dormancy-Related Phenotype of *Mycobacterium tuberculosis* . MBio 5, e01125-14. doi: 10.1128/mBio.01125-14 24846381 PMC4030484

[B49] Santucci P. Bouzid F. Smichi N. Poncin I. Kremer L. De Chastellier C.. (2016). Experimental Models of Foamy Macrophages and Approaches for Dissecting the Mechanisms of Lipid Accumulation and Consumption During Dormancy and Reactivation of Tuberculosis. Front. Cell. Infect. Microbiol. 6. doi: 10.3389/fcimb.2016.00122 PMC5054039 27774438

[B50] Sawers R. G. Falke D. Fischer M. (2016). Oxygen and Nitrate Respiration in Streptomyces coelicolor A3(2). Adv. Microb. Physiol. 68, 1–40. doi: 10.1016/bs.ampbs.2016.02.004 27134020

[B51] Schnappinger D. Ehrt S. Voskuil M. I. Liu Y. Mangan J. A. Monahan I. M.. (2003). Transcriptional Adaptation of *Mycobacterium tuberculosis* Within Macrophages: Insights Into the Phagosomal Environment. J. Exp. Med. 198, 693–704. doi: 10.1084/jem.20030846 12953091 PMC2194186

[B52] Solans L. Aguilo N. Samper S. Pawlik A. Frigui W. Martin C.. (2014). A Specific Polymorphism in *Mycobacterium tuberculosis* H37Rv Causes Differential ESAT-6 Expression and Identifies WhiB6 as a Novel ESX-1 Component. Infect. Immun. 82, 3446–3456. doi: 10.1128/IAI.01824-14 24891105 PMC4136221

[B53] Soto-Ramirez M. D. Aguilar-Ayala D. A. Garcia-Morales L. Rodriguez-Peredo S. M. Badillo-Lopez C. Rios-Muñiz D. E.. (2017). Cholesterol Plays a Larger Role During *Mycobacterium tuberculosis In Vitro* Dormancy and Reactivation Than Previously Suspected. Tuberculosis 103, 1–9. doi: 10.1016/j.tube.2016.12.004 28237027

[B54] Starck J. Källenius G. Marklund B.-I. Andersson D. I. Akerlund T. Åkerlund T. (2004). Comparative Proteome Analysis of *Mycobacterium tuberculosis* Grown Under Aerobic and Anaerobic Conditions. Microbiology 150, 3821–3829. doi: 10.1099/mic.0.27284-0 15528667

[B55] Tamburini B. Badami G. D. Azgomi M. S. Dieli F. La Manna M. P. Caccamo N. (2021). Role of Hematopoietic Cells in *Mycobacterium tuberculosis* Infection. Tuberculosis 130, 102109. doi: 10.1016/j.tube.2021.102109 34315045

[B56] Toossi Z. Gogate P. Shiratsuchi H. Young T. Ellner J. J. (1995). Enhanced Production of TGF-Beta by Blood Monocytes From Patients With Active Tuberculosis and Presence of TGF-Beta in Tuberculous Granulomatous Lung Lesions. J. Immunol. 154, 465–473. 7995958

[B57] Tripathi P. Singh L. K. Kumari S. Hakiem O. R. Batra J. K. (2020). ClpB is an Essential Stress Regulator of *Mycobacterium tuberculosis* and Endows Survival Advantage to Dormant Bacilli. Int. J. Med. Microbiol. 310, 151402. doi: 10.1016/j.ijmm.2020.151402 32014406

[B58] Turner R. J. Aharonowitz Y. Weiner J. H. Taylor D. E. (2001). Glutathione is a Target in Tellurite Toxicity and is Protected by Tellurite Resistance Determinants in *Escherichia Coli* . Can. J. Microbiol. 47, 33–40. doi: 10.1139/cjm-47-1-33 15049447

[B59] Waldbauer J. R. Rodrigue S. Coleman M. L. Chisholm S. W. (2012). Transcriptome and Proteome Dynamics of a Light-Dark Synchronized Bacterial Cell Cycle. PloS One 7, e43432. doi: 10.1371/journal.pone.0043432 22952681 PMC3430701

[B60] WHO (2021). Global Tuberculosis Report 2021 (Geneva: World Health Organization. WHO). Available at: http://apps.who.int/iris. Licence CC BY-NC-SA 3.0 IGO.

[B61] Wu M. Aung H. Hirsch C. S. Toossi Z. (2012). Inhibition of *Mycobacterium tuberculosis*-Induced Signalling by Transforming Growth Factor-β in Human Mononuclear Phagocytes. Scand. J. Immunol. 75, 301–304. doi: 10.1111/j.1365-3083.2011.02668.x 22150316 PMC3279592

