# Supplementary material for: The Lack of the TetR-Like Repressor Gene BCG_2177c (Rv2160A) May Help Mycobacteria Overcome Intracellular Redox Stress and Survive Longer Inside Macrophages When Surrounded by a Lipid Environment
Source: Front Cell Infect Microbiol. 2022 Jul 7;12:907890. doi: 10.3389/fcimb.2022.907890 (PMC9301340; doi:10.3389/fcimb.2022.907890)
Supplement: Supplementary file 1 [file Table_1.docx]

**Table S1. Primers used in this study for RT-qPCR**

| Gen ID | *Mtb* gen  ID (Rv) | Sequence 5'-3' | Amplicon size |
| --- | --- | --- | --- |
| *BCG_RS10340* | NA | F: TGCTCTCTAGAGCCCTGTCG  R: TCCGAGTTCTTCACGAAGGC | 91 |
| *BCG_RS02000* | *Rv0351 (Hsp70)* | F: TTCGCCAACTACCGTAAGCG  R: CGTCCAGTACACCCAGCAAT | 97 |
| *BCG_RS02945* | *Rv0532* | F: GTCTGGGTGGTGATGGTGG  R: CACCGAATCGAGCATCCACT | 90 |
| *BCG_RS17585* | *Rv3350c* | F: CCGGTCCCCGAATTGAAGAA  R: CAACCACCTGACGGGTATCG | 102 |
| *BCG_RS13040* | *Rv2504c* | F: CGAACTCCTTGTTCTCCCCC  R: GGAATCGACGGTGTTGGTCT | 91 |
| *BCG_RS20155* | *Rv3862c* | F: TTCGGGAATTACGACCCCTG  R: CGAGGCCAAGACCCTGTG | 109 |
| *BCG_RS10355* | *Rv1994c* | F: CTACCTGTCATCTCGACCGC  R: GTTGGTCCAGGTCGTTCTCG | 90 |
| *BCG_RS09005* | *Rv1706c* | F: CGTTACGTGAAAGGTTCGGC  R: GTTCTGCTCACCGCATTTCG | 102 |
| *BCG_RS17680* | *Rv3369* | F: TGAGCGTCGAACTGACACAA  R: TCGAAGTAGAACCACACCAGC | 118 |
| *BCG_RS09170* | NA | F: ACCGTCTCGCGGTAGTTGTA  R: TACCACCGTCAACGACAAGG | 120 |

Corresponding Rv was obtained from KEGG Data base <https://www.genome.jp/kegg/kegg2.html>. NA, no-orthologs found in *M. tuberculosis* H37Rv genome.
