# Supplementary material for: The Lack of the TetR-Like Repressor Gene BCG_2177c (Rv2160A) May Help Mycobacteria Overcome Intracellular Redox Stress and Survive Longer Inside Macrophages When Surrounded by a Lipid Environment
Source: Front Cell Infect Microbiol. 2022 Jul 7;12:907890. doi: 10.3389/fcimb.2022.907890 (PMC9301340; doi:10.3389/fcimb.2022.907890)
Supplement: Supplementary file 2 [file Table_2.docx]

**Table S2. Global gene expression (number of active genes) of wtBCG and mtBCG under different environmental conditions.**

| Functional Categories | BCG Ref-seq | BED | MED | BEL | MEL | BSD | MSD | BSL | MSL |
| --- | --- | --- | --- | --- | --- | --- | --- | --- | --- |
| Amino Acids and Derivatives | 155 | 129 | 152 | 151 | 144 | 142 | 148 | 152 | 144 |
| Carbohydrates | 146 | 124 | 140 | 141 | 135 | 131 | 138 | 140 | 130 |
| Cell Division and Cell Cycle | 16 | 15 | 16 | 16 | 15 | 16 | 16 | 16 | 16 |
| Cell Wall and Capsule | 49 | 40 | 48 | 48 | 46 | 44 | 45 | 49 | 44 |
| Cofactors, Vitamins, Prosthetic Groups, Pigments | 207 | 176 | 202 | 204 | 196 | 191 | 197 | 204 | 192 |
| DNA Metabolism | 65 | 53 | 61 | 63 | 61 | 57 | 61 | 63 | 59 |
| Dormancy and Sporulation | 1 | 1 | 1 | 1 | 1 | 1 | 1 | 1 | 1 |
| Fatty Acids, Lipids, and Isoprenoids | 117 | 98 | 114 | 114 | 109 | 107 | 108 | 116 | 108 |
| Functionally Unknow | 2535 | 2529 | 2408 | 2419 | 2317 | 2260 | 2341 | 2423 | 2265 |
| Iron acquisition and Metabolism | 2 | 1 | 2 | 2 | 2 | 1 | 2 | 2 | 2 |
| Membrane Transport | 24 | 20 | 23 | 24 | 23 | 22 | 23 | 23 | 22 |
| Metabolism of Aromatic Compounds | 7 | 6 | 7 | 6 | 6 | 6 | 7 | 6 | 6 |
| Miscellaneous | 32 | 29 | 31 | 30 | 31 | 29 | 31 | 32 | 30 |
| Motility and Chemotaxis | 1 | 1 | 1 | 1 | 1 | 1 | 1 | 1 | 1 |
| Nitrogen Metabolism | 33 | 28 | 32 | 33 | 30 | 29 | 32 | 32 | 31 |
| Nucleosides and Nucleotides | 61 | 50 | 60 | 61 | 58 | 56 | 59 | 61 | 57 |
| Phages, Prophages, Transposable elements, Plasmids | 1 | 1 | 1 | 1 | 1 | 1 | 1 | 1 | 1 |
| Phosphorus Metabolism | 20 | 18 | 20 | 20 | 20 | 18 | 19 | 20 | 20 |
| Potassium metabolism | 8 | 8 | 8 | 8 | 8 | 8 | 8 | 8 | 8 |
| Protein Metabolism | 174 | 147 | 166 | 165 | 164 | 161 | 164 | 167 | 161 |
| Regulation and Cell signaling | 50 | 38 | 47 | 47 | 45 | 46 | 46 | 48 | 43 |
| Respiration | 68 | 60 | 64 | 66 | 62 | 63 | 63 | 67 | 62 |
| RNA Metabolism | 56 | 53 | 56 | 56 | 56 | 54 | 56 | 56 | 55 |
| Secondary Metabolism | 2 | 2 | 2 | 2 | 2 | 2 | 2 | 2 | 2 |
| Stress Response | 50 | 43 | 49 | 49 | 47 | 47 | 47 | 49 | 45 |
| Sulfur Metabolism | 13 | 8 | 11 | 11 | 11 | 10 | 10 | 12 | 11 |
| Virulence, Disease and Defense | 74 | 63 | 71 | 70 | 67 | 66 | 68 | 72 | 67 |
| **Total** | **3967** | **3735** | **3786** | **3803** | **3651** | **3564** | **3688** | **3815** | **3578** |

**wtBCG,** wild-type *Mycobacterium bovis* BCG Pasteur 1173P2**; mtBCG,** a mutant strain of *M. bovis* BCG obtained by transposition of the *Tngfp* in the *BCG_2177c* gen, that codes for a putative TetR repressor; **BED**, wtBCG cultured at exponential phase in dextrose; **BEL**, wtBCG cultured at exponential phase in lipids; **MED**, mtBCG cultured at exponential phase in dextrose; **MEL**, mtBCG cultured at exponential phase in lipids; **BSD**, wtBCG cultured at stationary phase in dextrose; **BSL**, wtBCG cultured at stationary phase in lipids; **MSD**, mtBCG cultured at stationary phase in dextrose; **MSL**, mtBCG cultured at stationary phase in lipids.
